# Diversity of Pico- to Mesoplankton along the 2000 km Salinity Gradient of the Baltic Sea

**DOI:** 10.3389/fmicb.2016.00679

**Published:** 2016-05-12

**Authors:** Yue O. O. Hu, Bengt Karlson, Sophie Charvet, Anders F. Andersson

**Affiliations:** ^1^Science for Life Laboratory, Division of Gene Technology, School of Biotechnology, KTH Royal Institute of TechnologyStockholm, Sweden; ^2^Oceanography, Research & Development, Swedish Meteorological and Hydrological InstituteGothenburg, Sweden; ^3^Leibniz Institute for Baltic Sea Research WarnemündeRostock, Germany

**Keywords:** Baltic Sea, protists, bacterioplankton, brackish, metabarcoding, marine microbiology, microbial ecology

## Abstract

Microbial plankton form the productive base of both marine and freshwater ecosystems and are key drivers of global biogeochemical cycles of carbon and nutrients. Plankton diversity is immense with representations from all major phyla within the three domains of life. So far, plankton monitoring has mainly been based on microscopic identification, which has limited sensitivity and reproducibility, not least because of the numerical majority of plankton being unidentifiable under the light microscope. High-throughput sequencing of taxonomic marker genes offers a means to identify taxa inaccessible by traditional methods; thus, recent studies have unveiled an extensive previously unknown diversity of plankton. Here, we conducted ultra-deep Illumina sequencing (average 10^5^ sequences/sample) of rRNA gene amplicons of surface water eukaryotic and bacterial plankton communities sampled in summer along a 2000 km transect following the salinity gradient of the Baltic Sea. Community composition was strongly correlated with salinity for both bacterial and eukaryotic plankton assemblages, highlighting the importance of salinity for structuring the biodiversity within this ecosystem. In contrast, no clear trends in alpha-diversity for bacterial or eukaryotic communities could be detected along the transect. The distribution of major planktonic taxa followed expected patterns as observed in monitoring programs, but groups novel to the Baltic Sea were also identified, such as relatives to the coccolithophore *Emiliana huxleyi* detected in the northern Baltic Sea. This study provides the first ultra-deep sequencing-based survey on eukaryotic and bacterial plankton biogeography in the Baltic Sea.

## Introduction

The Baltic Sea is the world's second largest brackish water body. It is connected to the North Sea by several narrow straits. A horizontal salinity gradient ranging from 2 to 30 traverses the Baltic Sea and parts of the North Sea in a Northeast–Southwest (NE-SW) direction. This gradient is maintained by sporadic inflows of saline deep water in the south, in combination with freshwater input from rivers. A strong halocline at 60–80 m depth restricts vertical mixing in the Baltic Proper, and large areas of the deeper waters are hypoxic or anoxic (Carstensen et al., 2014). The intermediate salinity and subarctic conditions constrain the macrobial biodiversity in the Baltic Sea, and its multicellular species are fewer and typically genetically impoverished compared to the populations of the NE Atlantic (Johannesson and André, 2006). In contrast, bacterioplankton diversity does not appear to decrease at intermediate salinities (Herlemann et al., 2011), and for protists it has even been suggested that species richness is actually highest in the horohalinicum region (salinity 5–8; Telesh et al., 2011a, 2013).

Unicellular plankton play key roles in global biogeochemical cycles and form the basis of marine food webs (Falkowski et al., 2008). In the Baltic Sea, diatoms and dinoflagellates (>10 μm) dominate primary production in spring, while smaller eukaryotic phytoplankton and cyanobacteria (<3 μm) dominate primary production over the rest of the year (Johansson et al., 2004). Heterotrophic bacteria play key roles in taking up dissolved organic matter stemming from primary production within the ecosystem and from terrestrial runoff-water (Azam and Malfatti, 2007). Bacteria also mediate most transformations in the cycling of nitrogen, phosphorus, trace metals, and other nutrients (Falkowski et al., 2008). Energy and nutrients are propagated to higher trophic levels through grazing. Large phytoplankton are primarily grazed upon by mesozooplankton (e.g., copepods) but also by micro-zooplankton, i.e., heterotrophic protists. The current view is that picoplankton (bacteria and picoeukaryotes; 0.2–2 μm) are predated upon by nanoplankton (nanoflagellates and small ciliates; 2–20 μm), while the nanoplankton are eaten by larger zooplankton (copepods, rotifers, larger ciliates and dinoflagellates) (Samuelsson et al., 2006). The grazing on heterotrophic bacteria brings the organic matter leaked by phytoplankton during photosynthesis back to the traditional food chain, closing what is called the microbial loop (Azam et al., 1983). Finally, the larger zooplankton are predated upon by fish; in the pelagic Baltic Sea, mainly by sprat and herring. In this way, all elements of the pelagic ecosystem are interconnected, leading to cascade effects between different levels of the food web. For instance loss of top predators could be propagated all the way down to affect picoplankton community composition and function (Casini et al., 2009).

Monitoring of phyto- and zooplankton serves to estimate the environmental state and to detect changes regarding eutrophication, biodiversity, harmful algal blooms etc. This is today largely based on microscopic techniques (Hällfors, 2004; Olenina et al., 2006), which are time-consuming (~4 h/sample) and rely heavily on the skills of taxonomists. Cross-comparison between datasets analyzed by different taxonomists may therefore be problematic. The smallest plankton organisms, i.e., the pico- and nanoplankton (0.2–2 and 2–20 μm), and larger organisms that lack distinctive morphological features are difficult to identify using light microscopy. Therefore, these most abundant organisms are most often simply termed “Unicells” or “Flagellates” since they cannot be taxonomically identified. Hence, there is a need for faster, more robust novel methods that can cover a larger fraction of planktonic diversity.

Due to the recent developments of high-throughput sequencing techniques, genetic methods are a promising avenue for monitoring biological diversity. Genetic barcoding consists of taxonomically assigning a specimen based on sequencing a short standardized DNA fragment (barcode) and by matching this to a reference library of sequences of known taxonomy. By metabarcoding, the approach is extended to a community of individuals of different species. Metabarcoding has been extensively used for assessing both prokaryotic and eukaryotic plankton diversity and biogeography (Sogin et al., 2006; Amaral-Zettler et al., 2009; de Vargas et al., 2015). A number of metabarcoding studies have investigated diversity and spatiotemporal distribution patterns of prokaryotes in the Baltic Sea (Andersson et al., 2010; Herlemann et al., 2011; Dupont et al., 2014; Lindh et al., 2015), however, the methodology has not yet been used to map eukaryotic plankton in this sea.

Here, we have conducted ultra-deep Illumina sequencing of surface-water bacterial and eukaryotic plankton communities sampled in the summer of 2013 along a 2000 km transect following the salinity gradient of the Baltic Sea. The aim of the study is to give a comprehensive view of plankton diversity across the salinity gradient, and to investigate how microbial alpha- and beta-diversity is structured by salinity. To our knowledge, this is the first high-throughput sequencing-based investigation of eukaryotic plankton biogeography in the Baltic Sea and we combined it with a renewed description of the bacterial diversity highlighted by previous studies (Herlemann et al., 2011; Dupont et al., 2014).

## Materials and methods

### Sampling

Twenty-one water samples were collected in the Kattegat, the Baltic Proper and the Gulf of Bothnia using a FerryBox system installed in the ship TransPaper during 13th–19th of July 2013. The ship followed the route: Gothenburg (Sweden)—Kemi (Finland)—Oulu (Finland)—Lübeck (Germany)—Gothenburg. The FerryBox system consists of a pump with a water inlet at 3 m depth, a circuit of multiple sensors for temperature, conductivity, chlorophyll and phycocyanin fluorescence, turbidity, and oxygen as well as automated water sampling devices. A detailed description of the FerryBox system is found in Karlson et al. (in press). Manual water sampling for DNA analysis was carried out both on the Northward and Southward legs. Approximately, 10 L of seawater were collected in a polycarbonate carboy. Subsamples of 200–500 mL were filtered onto 0.22 μm pore-size mixed cellulose ester membrane filters (Merck Millipore co., Cat. No. GSWP04700) to capture plankton. The filters were frozen in liquid nitrogen on board and kept at −20 to −80°C until DNA extraction.

### DNA extraction, PCR amplification and sequencing

Genomic DNA was extracted using the PowerWater® DNA isolation kit (MO-BIO Laboratories Inc, Carlsbad CA, USA) following the instructions provided by the manufacturer. The V3-V4 regions of bacterial 16S rRNA genes were PCR amplified with primers 341F (CCTACGGGNGGCWGCAG) and 805R (GACTACHVGGGTATCTAATCC) (Herlemann et al., 2011), and the V4–V5 regions of eukaryotic 18S rRNA genes were amplified with primers 574*F (CGGTAAYTCCAGCTCYV and 1132R, CCGTCAATTHCTTYAAR; Hugerth et al., 2014a), using KAPA HiFi HotStart ReadyMix (2X) (KAPA Biosystems, Kit Code KK2602). A two step PCR procedure was applied (Hugerth et al., 2014a), with 35 (25 + 10) and 38 (28 + 10) PCR cycles for 16 and 18S, respectively. Between the first and second PCR, and prior to pooling libraries, amplicons were purified with 8.8% PEG 6000 (Polyethylene Glycol 6000) (Merck Millipore co., Cat. No. 528877-1KG) precipitation buffer and CA beads (carboxylic acid-coated superparamagnetic beads) (Dynabeads® MyOne™ Carboxylic Acid, Cat. No. 65012; Lundin et al., 2010). Agilent 2100 Bioanalyzer (Agilent, Technologies, DNA 1000 LabChip kit) and Qubit® 2.0 Fluorometer (Invitrogen, Qubit-IT™ dsDNA HS Assay kit) were used for checking the amplicon fragment sizes and quantification. Equimolar amounts of indexed samples were mixed and sequenced with Illumina MiSeq (Illumina Inc, USA) at NGI/Scilifelab Stockholm. The sequencing reads have been submitted to the European Nucleotide Archive (ENA) under accession numbers PRJEB12362.

### Sequence processing

The OTU table building procedures followed https://github.com/EnvGen/Tutorials, using USEARCH for quality trimming and OTU-clustering (Edgar, 2010), but with some optimizations. The primers were trimmed from forward and reverse reads with FastX (Pearson et al., 1997), as well as 40 bases in the 3′ end of the reverse reads (these displayed lower quality than the forward reads). This resulted in average Phred scores (*Q*-values) of every position in the trimmed reads to be >20. In order to avoid generating artificial OTUs while assigning as many reads as possible, we used more stringent criteria when generating OTU-centroid sequences than in the mapping (assigning step). For the centroid generation step, 3′-ends were further trimmed such that all remaining bases had Q ≥ 25. Forward and reverse reads were merged, and merged pairs that were shorter than 300 bp, had >3 mismatches, or overlapped by <100 bp were discarded. Remaining merged reads were clustered into OTUs using 99% identity level. For processing reads for the assigning step, the same procedure was applied, but with Q20 as the quality trimming parameter. The trimmed reads were mapped to the OTU centroids to build the 16S OTU table. 16S OTUs (their centroid sequences) were classified with SINA-1.2.13 using the SILVA 119 SSU database (Altschul et al., 1997; Quast et al., 2013; Decelle et al., 2015).

The 18S OTU table was built similarly to the 16S OTU table, but the overlap between read pairs was not sufficient for merging, so each pair was concatenated rather than merged. Primers were trimmed from forward and reverse reads, as well as 44 bases in the 3′ end of the reverse reads, resulting in average Phred scores of every position to be >20. After trimming, forward and reverse reads were concatenated prior to OTU clustering. For the taxonomic classification, OTU centroid sequences were split at the concatenation position, and the forward and reverse counterparts were separately BLAST (Altschul et al., 1997) searched against the PR2 database (based on GenBank 203–October 2014; Guillou et al., 2013). BLAST results were filtered such that only alignments of at least 250 and 210 bp in length (90% of the length of reads), for the forward and reverse read, respectively, were kept. Then, the highest taxonomic level was found where the forward and reverse read aligned to the same database sequence, with alignment identities exceeding the taxonomic level-specific cutoff. If just one database sequence was hit, the OTU inherited the taxonomy of this sequence. If multiple database sequences were hit, the bit-scores of the forward and reverse reads to each hit were summed, and the hits having at least 95% of the best sum of bit scores were selected, and the most detailed consensus taxonomy was built from these hits and assigned to the OTU. The taxonomic-level specific identity cutoffs were as follows: kingdom to family level (PR2 level 1–6): 90%; genus level (PR2 level 7): 97%, species level (PR2 level 8): 99%. These cutoffs were determined by running an *in-silico* experiment where artificial reads were generated from 1000 randomly selected PR2 sequences, and classified against the remaining sequences in PR2, using a range of identity cutoffs at each taxonomic level. The selected cutoffs were good compromises between classification accuracy (precision) and percentage of sequences classified (sensitivity), and these were for the simulation as follows: kingdom to family level: precision >95%, sensitivity >85%; genus level: precision 78%, sensitivity 42%; species level: precision 42%, sensitivity 38%. In addition to this automatic taxonomic annotation procedure, for a subset of the individual OTUs (*n* = 30) that were of special interest (e.g., being of high relative abundance) but could not be automatically annotated to a satisfactory detailed level, manual annotations by online BLAST searches against the NCBI nt database were performed, in which case only hits with coverage and identity >99% where considered, and among these the most detailed consensus taxonomy was assigned to the OTU.

### Statistical analysis

All statistical analysis and plotting were conducted in R (www.r-project.org) using the R libraries: vegan (α-diversity; β-diversity; subsampling; Mantel test), rworldmap (geographic plotting), ape (principal coordinates analysis), cluster (hierarchical clustering), and pheatmap (heatmap). For calculating the *p*-value of the observed level of matching between the taxonomies of correlated 16 and 18S OTUs, we randomly shuffled the taxonomic annotations among the 18S OTUs that displayed significant correlations to 16S OTUs, and compared the taxonomic annotations between the chloroplast 16S OTUs and the 18S OTUs. The shuffling was redone 10^6^ (*N*) times and we counted how many times (*n*) 5 or more of the 6 chloroplast 16S OTUs were annotated to the same taxonomic class as the paired 18S OTU (i.e., the same or higher level of matching as the real data). The *p*-value was calculated as *n*/*N*.

## Results

### Trends in beta-diversity

**Figure 1:**
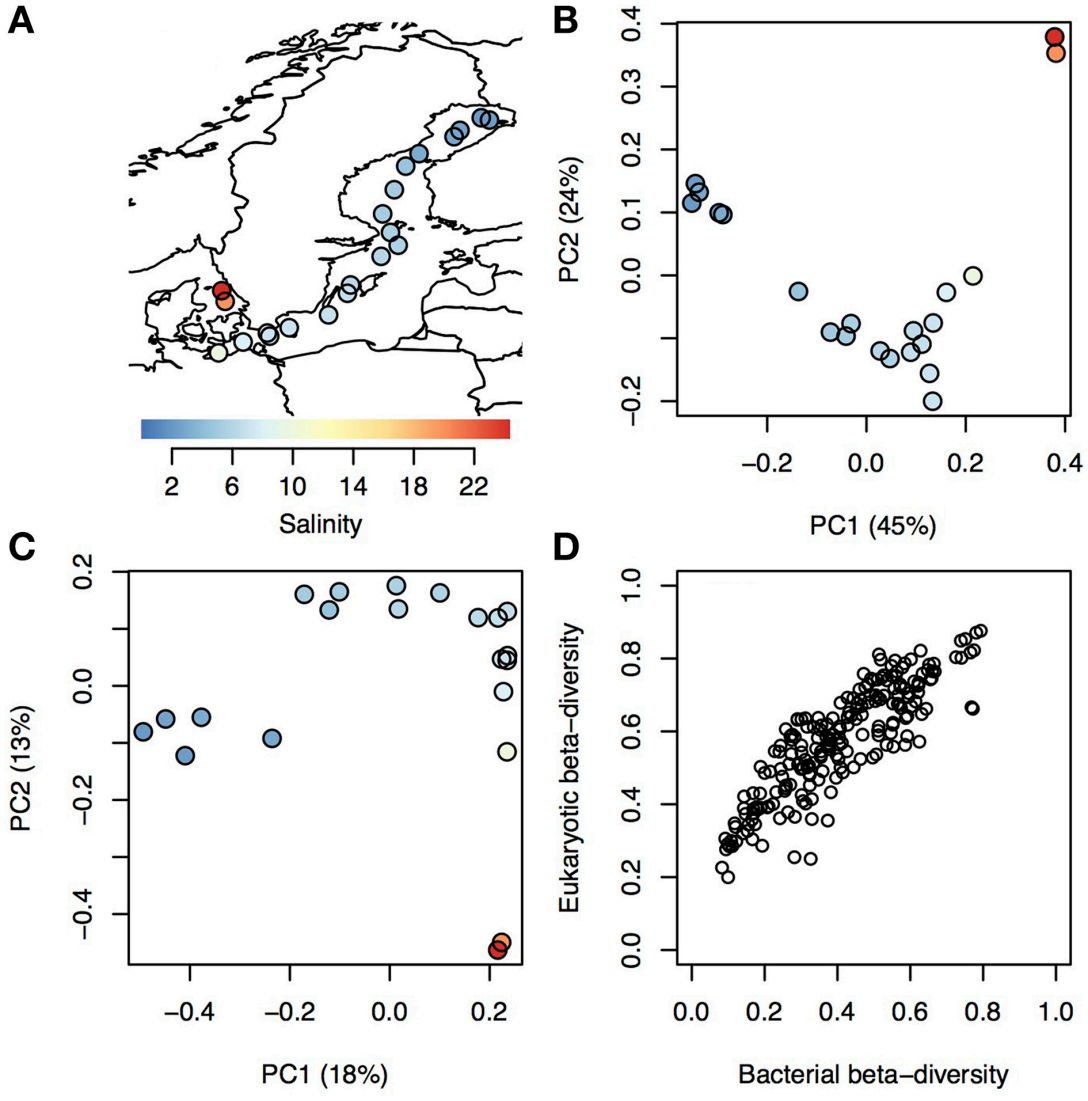
**Trends in bacterial and eukaryotic beta-diversity. (A)** Map showing sampling sites within Kattegat and the Baltic Sea, with salinity indicated by color. **(B–C)** Ordination of bacterial **(B)** and eukaryotic **(C)** communities using Principal Coordinate Analysis (PCoA) based on beta-diversity calculated using Spearman correlation of OTU frequencies. Samples are colored and sized according to **(A)**. Variation explained by the principal components (PCs) are given within parenthesis. PC1 was highly correlated with salinity for both bacterial and eukaryotic communities (Spearman ρ > 0.97 and *P* < 10^−5^ for both). **(D)** Pairwise dissimilarity score (beta-diversity) between all samples in the dataset, where the eukaryotic scores (y-axis) are plotted against the bacterial scores (x-axis) from the same samples.

On average 32,948 amplicons of bacterial (16S) and eukaryotic (18S) rRNA genes were sequenced from surface water samples from 21 stations within the Baltic Sea and Kattegat, collected in July 2013 (Figure 1A; stations are described in Supplementary Table 1 and seasonal variation in temperature and salinity of the sampled water bodies presented in Supplementary Figure 1). The sequences were clustered into 1243 and 1860 16 and 18S operational taxonomic units (OTUs), respectively (Supplementary Tables 2, 3). To investigate overall trends in community composition, Principal Coordinates Analysis (PCoA) was conducted on the bacterial and eukaryotic communities (Figure 1). Similar to what has been shown before in the Baltic Sea (Herlemann et al., 2011; Dupont et al., 2014), bacterial community composition changed gradually along the salinity gradient, with the first principal coordinate being highly correlated with salinity (Spearman rho = 0.99, *P* < 10^−5^; Figure 1B). Here we could also, for the first time, show a similar pattern for eukaryotic plankton in the Baltic (Spearman rho = 0.88, *P* < 10^−5^; Figure 1C). Likewise, hierarchical clustering grouped the samples according to their salinity, based on both bacterial and eukaryotic community composition (Supplementary Figure 2). Difference in salinity between samples was also directly correlated with difference in community composition (beta-diversity), for both bacterial and eukaryotic communities (Supplementary Figure 3). Difference in temperature, that to some extent covaries with salinity (Supplementary Table 1), was also correlated with beta-diversity, but the correlation was weaker (Supplementary Figure 3). Since both eukaryotic and bacterial communities correlated with salinity, it followed that beta-diversity of the two community types were correlated (Mantel test, Spearman rho = 0.86, *P* < 10^−6^; Figure 1D).

### Bacterial community composition

Our analysis confirmed the overall trends in bacterial taxonomic composition observed in previous studies, with an increase in Alpha- and Gammaproteobacteria and a decrease in Actinobacteria and Betaproteobacteria with increasing salinity levels (Herlemann et al., 2011; Dupont et al., 2014). Similar to what was found by Herlemann et al. (2011), Verrucomicrobia peaked in intermediate salinity levels, which here was also the case for Planctomycetes and Cyanobacteria (**Figure 3A**).

**Figure 2:**
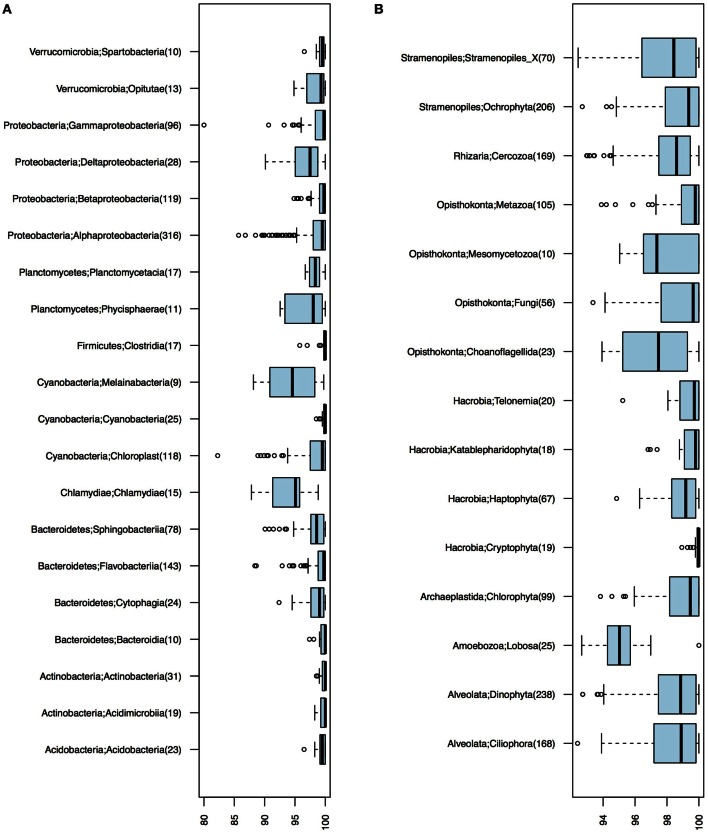
**Identity levels (%) between OTU centroid sequences and their best matching database sequence**. **(A)** Bacterial OTUs. **(B)** Eukaryotic OTUs. Number of OTUs included in each taxonomic group is given within parenthesis; only groups with ≥10 OTUs are shown.

Of the 1243 16S OTUs observed in the 21 samples, 1187 could be taxonomically classified at least to the class level. The majority (59%) of the OTU centroid sequences matched SILVA database reference sequences with >99% identity. Identity levels however varied substantially among taxa (Figure 2). Chlamydiae and the newly discovered non-photosynthetic sister clade to Cyanobacteria, the Melainabacteria (Di Rienzi et al., 2013), displayed notably low identity levels (Figure 2A).

**Figure 3:**
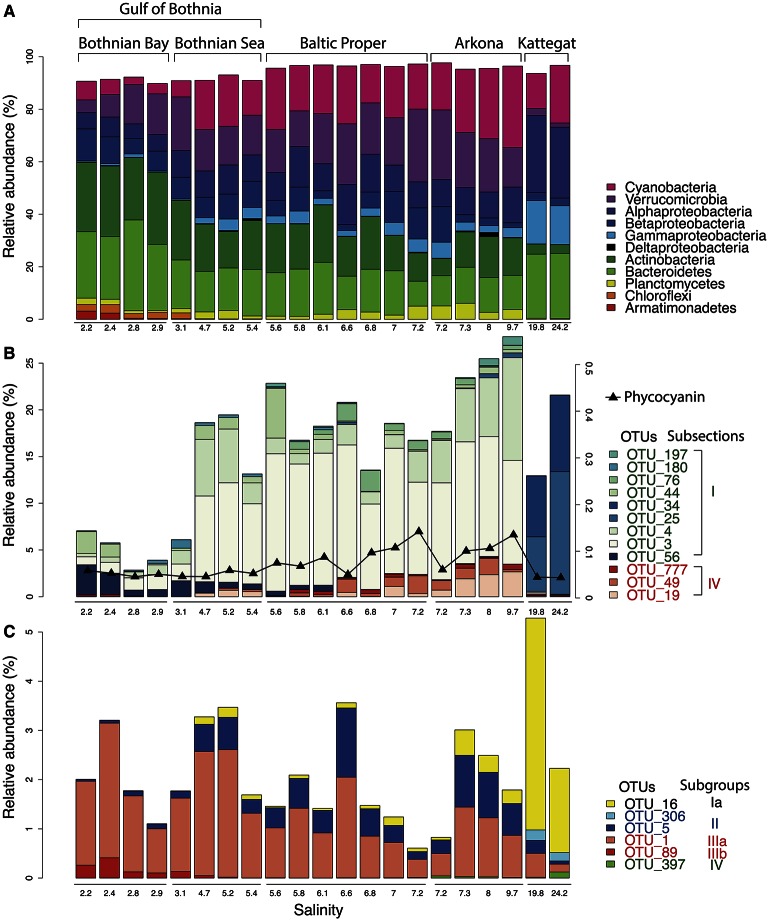
**Abundance distribution of major bacterial groups across the Baltic Sea salinity gradient**. **(A)** Major bacterial taxonomic groups (>0.1% mean abundance). **(B)** Dominant Cyanobacteria OTUs (>0.1% mean abundance). In Subsection I (picocyanobacteria), OTU_56 is a *Microcystis* (a freshwater species), remaining OTUs belong to *Synechococcus*. In Subsection IV (filamentous cyanobacteria), OTU_777, OTU_49 and OTU_19 belong to *Aphanizomenon, Nodularia,* and *Dolichospermum* (syn. *Anabaena*), respectively. The black curve indicates phycocyanin fluorescence (in arbitrary units). **(C)** Dominant SAR11 OTUs (>0.1% mean abundance).

The gradual shift in community composition observed at the phylum/class level was also evident within specific taxonomic groups, as exemplified by the Cyanobacteria and the Alphaproteobacteria cluster SAR11 in Figures 3B,C. The Cyanobacteria were represented by 26 OTUs, of which 12 had a mean abundance of >0.1% of reads, most of which were related to the picocyanobacteria *Synechococcus* (Figure 3B). OTUs associated to filamentous cyanobacteria (*Aphanizomenon, Nodularia*, and *Dolichospermum* (syn. *Anabaena*)) were also detected from the brackish region but in much lower abundances than the dominant *Synechococcus*, consistent with cyanobacterial cell numbers and biomass being dominated by picocyanobacteria in the Baltic Sea (Stal et al., 2003). Phycocyanin fluorescence, as recorded by the FerryBox system, correlated well with relative abundance of sequenced filamentous cyanobacteria (Spearman rho = 0.69, *P* < 10^−3^), in agreement with Seppälä et al. that observed a linear relation between biomass of filamentous cyanobacteria and phycocyanin fluorescence (Seppälä et al., 2007). The different *Synechococcus* populations showed clear differences in salinity preference, with OTU_25 and OTU_34 being dominant in Kattegat at more than 99.7% of cyanobacterial reads, while OTU_3 and OTU_4 dominated the Baltic Proper with on average 88% of cyanobacterial reads. OTU_3 was identical to the 16S sequence of a metagenome-assembled genome reconstructed from the Baltic Proper (BACL30; Hugerth et al., 2015) that possess the type IIB phycobilisome gene organization recently identified as being dominant in Baltic Sea picocyanobacteria (Larsson et al., 2014).

SAR11 is the most abundant bacterioplankton clade of the world's oceans, and is also found in brackish and fresh waters. It consists of several subclades that together span the candidate order Pelagibacterales (Grote et al., 2012). Eight OTUs were classified as SAR11 in our dataset, of which six had >10 reads (Figure 3C), representing five different subclades of SAR11 (Supplementary Figure 4 for a phylogenetic tree). An OTU (OTU_16) identical to *Pelagibacter ubique* (open ocean subclade Ia) dominated the marine samples but was also present in the brackish samples, while two OTUs belonging to subclade IIb (OTU_5) and IIIa (OTU_1) dominated the brackish region. Subclade IIIa is frequent in brackish waters and has previously been found in the Baltic Sea (Herlemann et al., 2011; Bergen et al., 2014; Dupont et al., 2014), while subclade II is mainly associated with coastal and mesopelagic waters (Carlson et al., 2009; Beman et al., 2011). Interestingly, contrary to previous studies (Bergen et al., 2014; Dupont et al., 2014), we also found an OTU representing the freshwater subclade IIIb (LD12) in the low-salinity Bothnian Bay.

The deep sequencing provided by the Illumina platform allowed clustering of reads with higher phylogenetic resolution than with earlier 454-data (99% identity; compared to 97% in the study by Herlemann et al., 2011) while still maintaining enough reads per OTU to accurately quantify them. This uncovered highly similar OTUs with distinct spatial distributions. Herlemann et al. (2011) found that the most abundant OTU was a Verrucomicrobia belonging to the class Spartobacteria, for which the genome was later reconstructed with metagenomics (Herlemann et al., 2013), and the name Candidatus *Spartobacteria baltica* was proposed. In the current dataset we obtained an OTU identical to *S. baltica* (OTU_2) that was among the most abundant OTUs in the Baltic Proper, but we also obtained closely related OTUs (>98% identity to OTU_2) with contrasting distribution patterns over the salinity gradient (Supplementary Figure 5).

### Eukaryotic community composition

**Figure 4:**
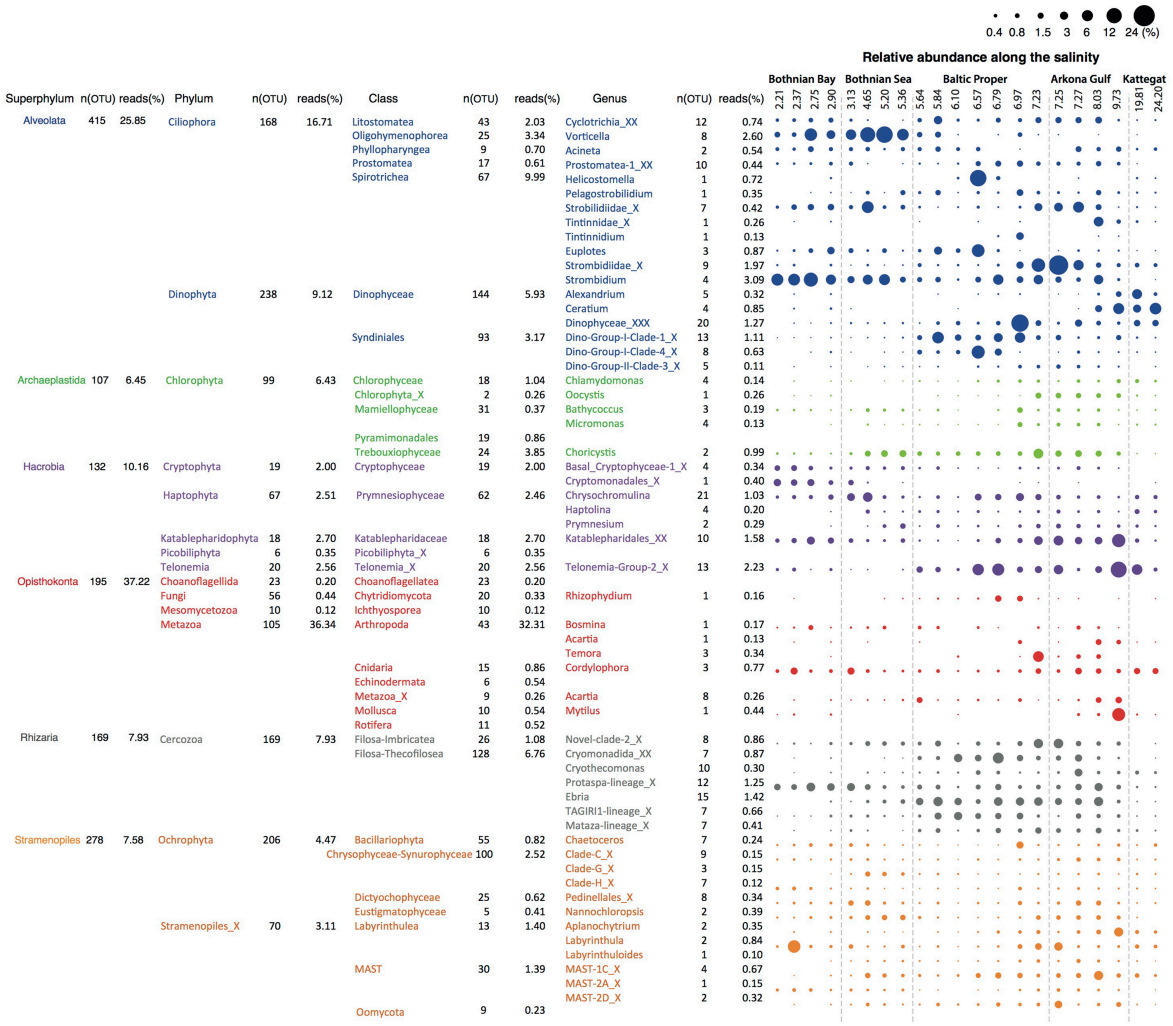
**Summary of detected eukaryotic taxonomic groups**. The number of detected OTUs and the mean abundance is given for each taxonomic group at the superphylum, phylum, class and genus level (corresponding to PR2 levels 2, 3, 4, and 7). Only groups displaying >0.1% mean abundance are shown. The dot plot illustrates the distribution of the annotated genera along the salinity gradient, with circle area corresponding to relative abundance.

We retrieved 18S rRNA gene reads corresponding to nine eukaryotic super-phyla, 24 phyla, 77 classes and 256 genera (see Figure 4 for details). Since no prefiltration was applied before the plankton were captured on filters, the eukaryotic organisms span a large size range, and include unicellular organisms (protists) as well as multicellular organisms (metazoa). The phyla Metazoa, Ciliophora, Dinophyta, Cercozoa, Chlorophyta, Katablepharidophyta, Stramenopiles_X, Ochrophyta, Telonemia, Haptophyta, and Cryptophyta each makes up >1%, and together comprise 94% of the total 18S sequence reads. Stramenopiles_X is a division used in the PR2 database and includes heterotrophic unicellular heterokonts, e.g., the Oomycota, MAST and Labyrinthula. Some samples were highly dominated by metazoan sequences (mainly copepods), likely due to the large number of rRNA genes in their genomes and the fact that these are multicellular organisms. Nevertheless, they never exceeded 70% of the sequences in a sample, leaving at least 17,226 sequences for description of the remaining community of unicellular protists.

Of the total 1860 18S OTUs, 283 could be attributed to a known genus or species, 309 could be assigned to PR2 phylogenetic clusters corresponding to the genera level, and 740 could only be classified to higher taxonomic levels. Remaining OTUs could not be classified using the PR2 database, but these corresponded to only 5.63% of the reads in the samples on average. As was observed for the bacteria, identities of closest-related database sequences varied substantially between clades (Figure 2B). Groups with unusually low similarity levels (median < 98%) were Amoebozoa, Choanoflagellida, and Mesomycetozoa.

### Eukaryotic phytoplankton

Recognizing that some protistan groups include phototrophic, mixotrophic and heterotrophic organisms, a division of the organisms in phytoplankton and heterotrophic plankton has been made. Among the dinoflagellates many are mixotrophic, i.e., they carry out photosynthesis but also feed on other organisms. Here mixotrophs are treated as the part of the phototrophic phytoplankton.

**Figure 5:**
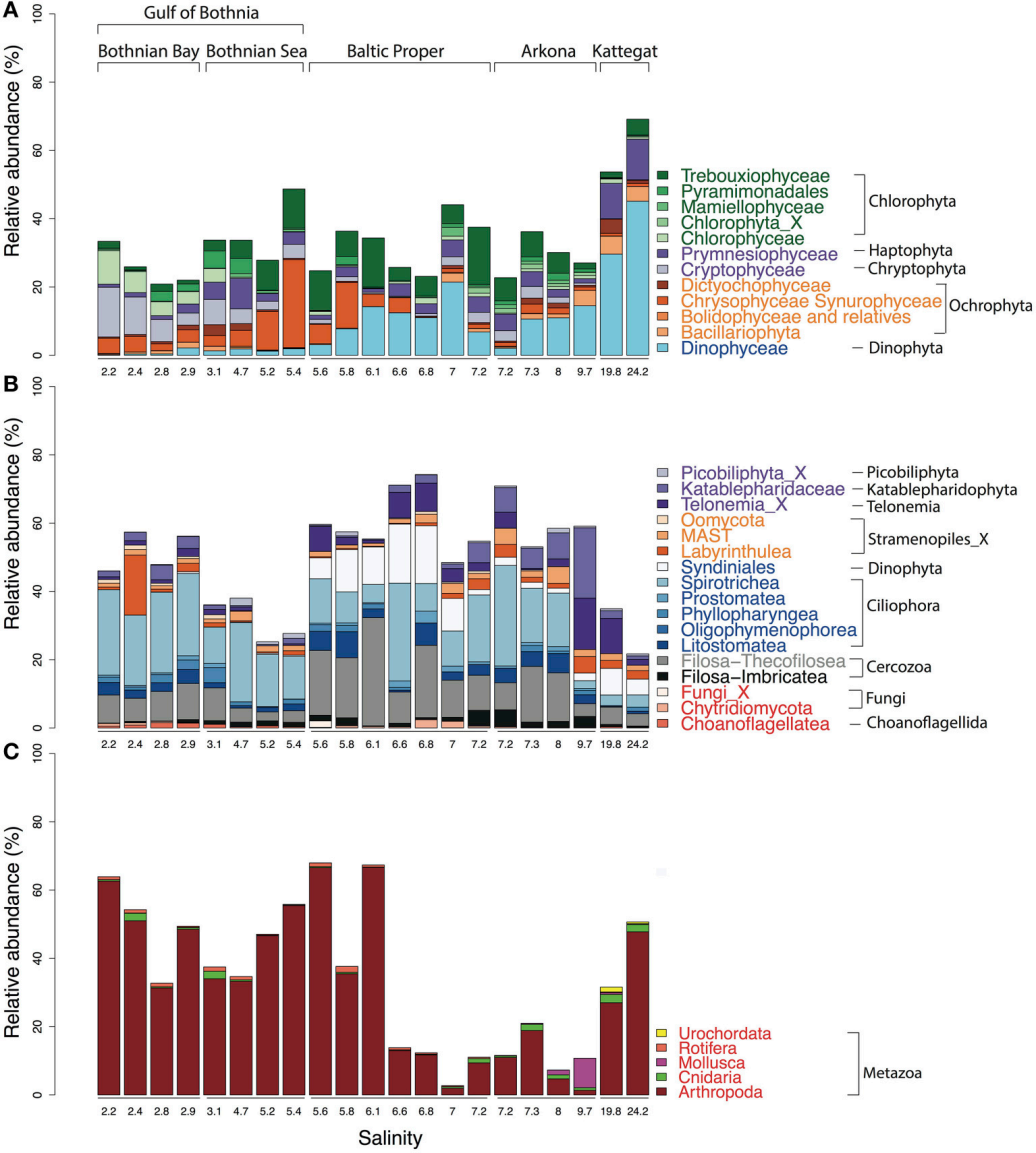
**Abundance distribution of major eukaryotic taxonomic classes of plankton across the Baltic Sea salinity gradient**. Classes displaying >1% mean abundance are shown and colored according to the superphylum they belong to. Green, purple, orange, blue, gray, and red represent Archaeplastida, Hacrobia, Stramenopiles (or Heterokonta), Alveolata, Rhizaria, and Opisthokonta, respectively. **(A)** Phytoplankton, here broadly defined as groups of organisms that include mainly phototrophic and mixotrophic representatives, although some groups, such as Dinophyta, also include organisms that are heterotrophic. **(B)** Heterotrophic plankton excluding metazoa. **(C)** Metazoa. Relative abundances in **(A,B)** indicate percentages of protist (i.e., non-metazoan) sequence reads; in **(C)** percentages of all eukaryotic sequence reads.

Microphytoplankton, made up of diatoms and dinoflagellates, are characteristic of the saline southern and western parts of the Baltic Sea and Kattegat, while smaller eukaryotic phytoplankton groups are more dominant in the northern Baltic Sea (Ojaveer et al., 2010). Pronounced seasonal fluctuations also prevail, with diatoms and dinoflagellates blooming in spring, and smaller eukaryotic phytoplankton and cyanobacteria dominating in summer (Johansson et al., 2004). Diatoms (Bacillariophyceae) and dinoflagellates (Dinophyceae) had higher relative abundances in the more saline regions of our survey (Figure 5A). However, although the samples were taken after the spring bloom, a substantial fraction of the reads from the Baltic Proper also broadly classified as dinoflagellates (Figure 5A). On average, dinoflagellates contributed to 10% of the reads. The relative proportion of dinoflagellates decreased from 50% to nearly 0% of the protist fraction, going from highest to lowest salinity (Figure 5A).

In the high salinity Kattegat, dominant dinoflagellate genera were *Alexandrium* and *Ceratium* of the order Gonyaulacales (Supplementary Figure 6B), while in the Baltic Proper, typical brackish dinoflagellates from the order Suessiales, such as *Biecheleria baltica* (OTU_25), were most abundant. Major OTUs of diatoms belonged to species often found in Baltic monitoring programs, such as the polar centric diatoms *Attheya longicornis* (OTU_144) and *Chaetoceros neogracile* (OTU_136), and the raphid diatom *Cylindrotheca closterium* (OTU_94). Interestingly, at the station south of Öland in the Baltic Proper (salinity 6.97), the Bacillariophyta and Dinophyceae were both dominated by OTUs (OTU_125 and OTU_32) that displayed highest similarity (99.7 and 98.1% identity) to sequences of uncultured diatoms and dinoflagellates from anoxic layers of the Framvaran Fjord in Norway (Behnke et al., 2010; Supplementary Figures 6A,B). Why these potentially anaerobic diatoms and dinoflagellates flourished in the surface water at this particular station remains to be explained.

Unlike the distribution pattern of dinoflagellates and diatoms, other phytoplankton, such as Chrysophyta, Chlorophyta, and Cryptophyta, were observed at lower salinities. The typical freshwater groups, Chrysophyceae-Synurophyceae (these two classes are combined in the PR2 database), were abundant in the surface water in the Gulf of Bothnia, peaking with 25% of protist reads at salinity 5.4 in the Bothnian Sea (Figure 5A). The dominant OTU (OTU_4) in this area belonged to *Uroglena* (100% identity), a common colony-forming genus, that made up nearly half of the phototrophic protist community reads. The green algae group, Chlorophyta, was found prevalent in the Baltic Proper and Gulf of Bothnia (Figures 4, 5A). In the Baltic Proper it was dominated by the genus *Choricystis* (class Trebouxiophyceae) (OTU_9), while in the Gulf of Bothnia two Chlorophyta freshwater OTUs (OTU_103: order Chlamydomonadales, OTU_65: *Monoraphidium convolutum*), together with one Cryptophyta freshwater species (OTU_56: *Rhodomonas minuta*) made up one third of the phytoplankton reads (Supplementary Figure 6C).

The ultra-deep sequencing also revealed organisms not easily identifiable with a microscope, such as picoeukaryotes of the class Mamiellophyceae within the Chlorophyta (Figure 4), from which we detected OTUs of the genera *Ostreococcus, Micromonas* and *Bathycoccus* (Supplementary Figure 6E). Though they never contributed with more than 2.5% of the protist reads, they displayed distinct distribution patterns along the salinity gradient. For example, *Ostreococcus*, the smallest known eukaryote, was associated with two OTUs that were separately abundant in Bothnian Sea (OTU_315) and Arkona Basin (OTU_464). Notably, the OTU that peaked in the Bothnian Sea was absent from the high salinity Kattegat region. This OTU belonged to *Ostreococcus* clade D according to its high similarity (1 deletion) to clade D strains isolated from the Mediterranean (Subirana et al., 2013), while OTU_464 belonged to clade C with its representative sequence being identical to 18S of the widespread *Ostreococcus tauri* (Keeling, 2007). Our results were consistent with a study on global distribution of *Ostreococcus* (Demir-Hilton et al., 2011), which reported that Clade D was only found in estuarine or brackish waters, while Clade C was also found in marine waters.

The most abundant Haptophyta OTUs were most closely related to Coccolithophorids (class Prymnesiophyceae), which are globally important phytoplankton with characteristic exoskeletons of calcium carbonate plates (coccoliths). They are found in large numbers throughout the ocean's euphotic zone but only one species, *Balaniger balticus*, has been described in the Baltic Sea (Thomsen and Oates, 1978). The sparsity of coccolithophorids in the Baltic has been attributed to undersaturation of calcium carbonate in winter, potentially resulting in dissolution of the coccoliths (Tyrrell et al., 2008). We found two coccolithophore-related OTUs (OTU_127 and OTU_2007) that reached a maximum of 4.2% of the protist reads in the high salinity samples in Kattegat. The representative sequences of these two OTUs showed 100% identity to both coccolithophore species *Emiliania huxleyi* and *Gephyrocapsa muellerae* from the family Noelaerhabdaceae, which, incidentally, have identical sequences (Bendif et al., 2015). Intriguingly, OTU_127 was also detected, though at much lower abundances, in the Gulf of Bothnia (Supplementary Figure 7). Here we also detected the non-calcifier *Isochrysis galbana* (OTU_585) that belong to the same order (Isochrysidales) as the coccolithophores. As far as we know, neither *Emiliania*/*Gephyrocapsa* or *Isochrysis* have been observed in the Baltic Sea before. Although OTU_127 dominated the prymnesiophytes in Kattegat, coccolithophores were not the predominant organisms from this group in the Baltic regions. Instead, order Prymnesiales was dominant with OTUs widely spread according to their salinity preferences (Figure 4). *Prymnesium*, a genus that include the potentially toxic spring-bloom forming species *Prymnesium polylepis* (Gorokhova et al., 2014), was present in all samples except those collected in the Bothnian Bay (Figure 4).

While the above data on eukaryotic phytoplankton are based on 18S rRNA gene amplicons, phytoplankton can potentially also be monitored using 16S sequencing of chloroplast sequences (Eiler et al., 2013; Lemieux et al., 2014). In line with the fact that our 16S primer pair matches a fair amount of chloroplast sequences (65% of chloroplast sequences in RDP v.11.4, Cole et al., 2014), 120 of the 16S OTUs, corresponding to on average 0.3% of the reads, were classified as chloroplasts by SILVA, and a subset of these (98 OTUs) could be further classified by PhytoREF (Decelle et al., 2015). Ideally, the 16 and 18S data should reveal the same trends across samples for the same clade or species. We tested this by first correlating relative counts at the class level. For the seven classes that had at least 1000 reads in total in each dataset (16 and 18S), six displayed significant correlations (*P* < 0.05; average Spearman correlation coefficient = 0.75). Comparing at a more detailed taxonomic level was complicated by the fact that the 16S OTUs lacked a detailed taxonomic annotation. Instead we correlated all 16S OTUs to all 18S OTUs and extracted the pairs displaying a Spearman rank order correlation coefficient of at least 0.8. Of the 304 pairs found, six included a 16S OTU classified as chloroplast. For five of these six pairs, the 16 and 18S OTU were classified to the same taxonomic class (four different classes; Supplementary Figure 8). The probability that this level of matching would occur by chance is extremely low (*P* < 10^−6^; see Methods), and hence these five pairs of OTUs likely represent the same species or genus. This demonstrates the robustness of the metabarcoding approach and indicates that this type of correlation analysis can be fruitful for linking chloroplast 16S sequences to their nuclear 18S counterparts.

### Eukaryotic heterotrophs

The detected heterotrophic protists mainly belonged to the phyla Ciliophora, Cercozoa, Dinophyta, Katablepharidophyta, and Telonemia (Figure 5B). Ciliates (Ciliophora) that carry out photosynthesis by using klepto-chloroplasts belong to the functional group phytoplankton but are treated among the heterotrophs is in this article. Heterotroph dominance varied between class Spirotrichea from the phylum Ciliophora, in the Gulf of Bothnia and the Southern Baltic, and class Filosa-Thecofilosea from the phylum Cercozoa, in the Northern Baltic Proper (Figure 5B). The abundance of both these classes dropped in the Kattegat, as well as in the “intermediate” Arkona sample (salinity 9.7).

Of the 173 OTUs classified as Ciliophora, 32 had a mean relative abundance >10^−3^. The most prominent OTUs belonged to the genus *Vorticella* (OTU_2) from class Oligohymenophorea and to *Strombidium* (OTU_5) from class Spirotrichea (Figure 5, Supplementary Figure 6F). Only two OTUs (OTU_425, OTU_666), that displayed rather low abundance, belonged to the genus *Mesodinium* (Mironova et al., 2009), normally abundant in the Baltic Sea. The low yields could be due to diurnal vertical migration, or that the *Mesodinium* population was present in thin layers that were not sampled (Sjöqvist and Lindholm, 2011). Cercozoa is a highly cryptic phylum that contains widespread protozoan omnivores and parasites with vague morphological characters (Cavalier-Smith, 1998; Bass et al., 2009; Weber et al., 2012). Of the 170 Cercozoa OTUs, 25 had a mean relative abundance >10^−3^ (Figure 5, Supplementary Figure 6G).

Other heterotrophic strategies, such as parasitism, also play essential roles in the microbial food web, but monitoring parasites or symbionts by microscopy can be difficult. Sequencing of marker genes can greatly facilitate depiction of the distribution pattern of these organisms. For instance, Syndiniales, a class of Dinophyta that are parasites on other dinoflagellates, were found where other dinoflagellates were abundant (Figures 5A,B). In total, 93 OTUs were found from three Syndiniales groups (group I, II, III) (Guillou et al., 2008). The dominant OTUs were mainly from group I (Figure 4), while group II was the most diverse with 56 OTUs.

In the Hacrobia division, three major heterotrophic classes, Picobiliphyta (Picozoa), Katablepharidaceae, and Telonemia, showed contrasting distribution patterns in the Baltic. The Picobiliphyta, initially described from cold polar waters (Lovejoy et al., 2006), and recently renamed as Picozoa (Seenivasan et al., 2013), is a highly diverse deep-branching clade, with representatives found in a variety of marine ecosystems (Not et al., 2007, 2009). In the present study, the dominant Picozoa OTU (OTU_44), possessing almost 80% of the Picozoa reads, was identical to the only cultured member of the Picozoa, *Picomonas judraskeda*, that feed on small organic particles (Seenivasan et al., 2013). These picoeukaryotes (2–5 μm length) have previously been enriched from brackish Baltic Sea water under dark incubations (Weber et al., 2012). Abundance of Picozoa peaked in the Bothnian Sea, while the larger Katablepharydophyta and Telonemia accumulated mainly in the Baltic Proper and the Arkona basin.

The superphylum Stramenopiles were represented by the Ochrophyta and Stramenopiles_X. The 70 Stramenopiles_X OTUs were mainly annotated to the classes Labyrinthulea, Oomycetes, and MAST. One of the samples from Bothnian Bay (salinity 2.4) displayed markedly higher abundance of Labyrinthulea than the others (Figure 5B). Class Labyrinthulea was highly dominated by an OTU (OTU_54) that was found in all Baltic regions with no apparent salinity preference. The representative sequence of OTU_54 was highly similar (99–100% identity) to endophytic Labyrinthula strains isolated from either mangrove leaves or eelgrass, which indicates that OTU_54 is an endophyte associated with plants. Unlike other sampling sites, the depth at this station is rather shallow (17-m), and directly to the east a region of shallow waters (10-m depth on average) extends to the Finnish coast. Hence, the high abundance of Labyrinthula here could be due to debris from benthic macro vegetation captured on the filter.

Of the 70 OTUs from Stramenopiles_X, 30 were annotated to the MAST lineage, a diverse group of pico- to micro-sized heterotrophic marine stramenopiles that was discovered by environmental sequencing only 10 years ago (Massana et al., 2004) and has not yet been cultured (Massana et al., 2006). Many studies have since then investigated the links between environmental factors and MAST lineages (Massana et al., 2004, 2014; Piwosz and Pernthaler, 2010; Lin et al., 2012; Thaler and Lovejoy, 2013). Here, seven MAST clades (“ribogroups”; Massana et al., 2014) were detected. The most prevalent were MAST-1 and -2 (Figure 4, Supplementary Figure 6H), which are commonly found in marine and coastal regions (Massana et al., 2004, 2006). To date, four subclades of MAST-1 are known (Massana et al., 2014), however, only MAST-1C was observed in our dataset, with five associated OTUs (Supplementary Figure 6H). Besides the most prevalent MAST clades (MAST-1C, -2D), MAST-4D and -6 were abundant in the brackish region (salinity 4–10). For MAST-6, all OTUs (OTU_145, 245, and 644) were missing in Bothnian Bay and Kattegat, which indicates these are brackish specialists. In the Gulf of Bothnia, MAST-2A, -3J, -12C, and -12_X displayed pronounced preference of low salinity (<4) (Supplementary Figure 6H). So far, only sequences from MAST-2 and -12 have been found in freshwater (Massana et al., 2014). The OTU (OTU_337) from MAST-3J that only appeared in the Bothnian Bay indicates that also this subclade contains freshwater members. In contrast, other MAST-3 subclades only appeared in the high salinity Kattegat samples.

**Figure 6:**
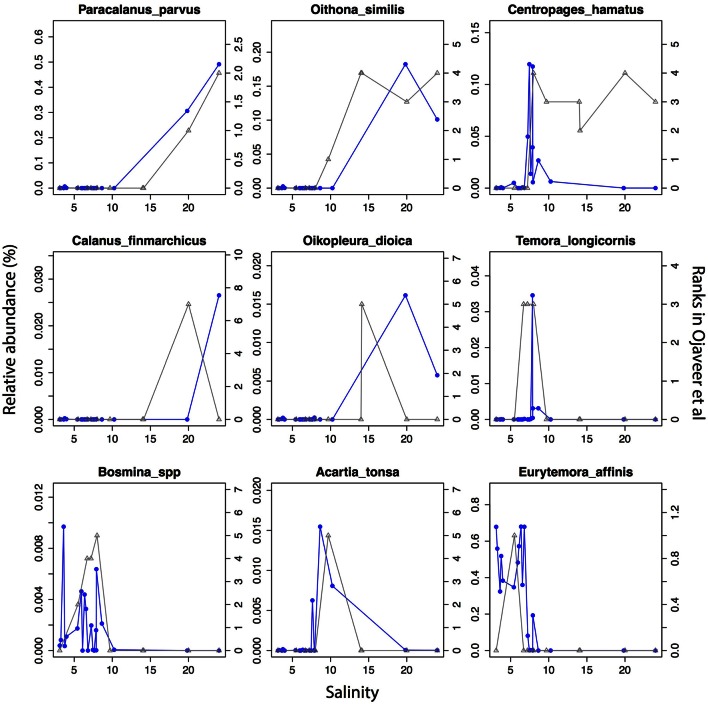
**Abundance distribution of dominant mesozooplankton (Metazoa) across the salinity gradient in comparison to historical monitoring data**. Data from Figure 1 and Table 3 of Ojaveer et al. (2010) is indicated with triangles connected by gray lines (right y-axis). Relative abundances of the OTUs for the corresponding species (when a species corresponds to multiple OTUs these were summed) are indicated with circles connected by blue lines (left y-axis).

The detected unikonts (superphylum Opisthokonta) in this study included Choanoflagellates, Mesomycetozoa, Fungi, and Metazoa, with 195 OTUs annotated in total. The most divergent and abundant group was Metazoa (Figure 5C) with 105 OTUs of which 15 had a mean relative abundance >10^−3^. This can be compared with 217 species of metazooplankton in the Baltic Sea based on zooplankton inventories (Telesh et al., 2009). A recent review of plankton diversity in the Baltic ranked 23 abundant mesozooplankton species in different subbasins based on monitoring data (Ojaveer et al., 2010). OTUs corresponding to these Metazoa species displayed remarkably similar distribution patterns (Figure 6). Unicellular unikonts (Choanoflagellates, Mesomycetozoa, Fungi) made up a limited fraction of protist reads (Figure 5B). The low abundance of choanoflagellates may be attributed to seasonal dynamics, since unlike other heterotrophic nanoflagellates, they have been reported as being sparse during summer in the Baltic Sea (Samuelsson et al., 2006).

### Trends in alpha-diversity along the salinity gradient

**Figure 7:**
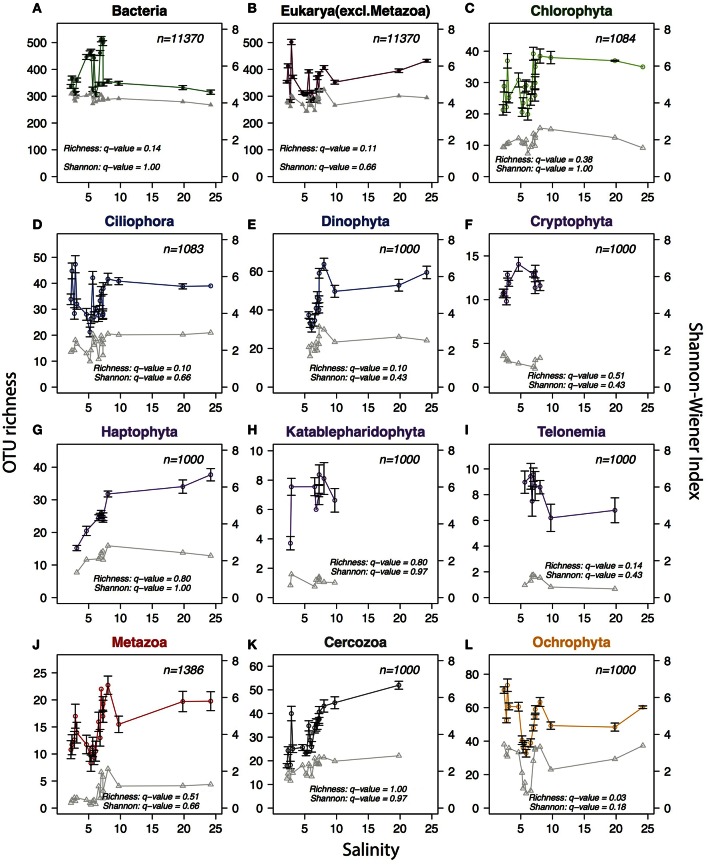
**Trends in planktonic alpha-diversity along the salinity gradient**. Circles connected by colored lines indicate observed OTU richness (left y-axes) and triangles connected by gray lines represent Shannon-Wiener diversity index (right y-axes). In order to remove biases due to variation in sequence depth, the same number of reads (indicated with *n* in each panel) were subsampled from each sample. The subsampling procedure was repeated 1000 times and the averages and standard deviations (error bars) were calculated. For Shannon-Wiener standard deviations were negligible and are not shown. **(A)** Total bacterial community. **(B)** Total protist community (non-metazoan eukaryotes). **(C–L)** Eukaryotic phyla with mean relative abundance >1%. For each phylum (*n*) reads were subsampled from reads belonging to the respective phylum, to avoid that diversity indices were biased by the relative abundance of the phylum within the sample. A minimum of 1000 reads were subsampled per phylum/sample; samples containing less than 1000 reads from the phylum were excluded. Phyla were colored according to superphylum in accordance with Figures 4, 5. *Q*-values indicate False Discovery Rate-adjusted p-values of Wilcoxon rank sum tests comparing Shannon-Wiener index and OTU richness in the horohalinicum samples (salinity 5–8; *n* ≤ 11) and the other samples (*n* ≤ 10).

The deep sequencing allowed us to investigate trends in alpha-diversity over the salinity gradient without the limitations associated with microscopic detections. Since alpha-diversity estimates are biased by sequencing depth (Lundin et al., 2012), the same number of reads (*n*) were subsampled from each sample, where *n* equals the lowest number of reads available among samples for the community or phylum analyzed. No clear trends in species richness (number of observed OTUs) or combined richness-evenness (Shannon-Wiener index) could be observed for either bacteria or protists at the total community level (Figure 7). When zooming-in on specific eukaryotic phyla (here *n* reads were sampled from within each phylum) no consistent trends were observed, but one of the phyla, Ochrophyta, appeared to have lower diversity in the intermediate salinity range. Comparing the species richness and Shannon index between the samples in the horohaliculum (salinity 5–8) with the other samples gave significant results only for Ochrophyta, that had significantly lower richness in the horohalinicum (Wilcoxon rank sum test, False Discovery Rate-adjusted *p* = 0.03; Figure 7).

## Discussion

The Baltic Sea has an exceptional long record of plankton monitoring, based primarily on microscopy, from the HELCOM-COMBINE monitoring program (HELCOM, 2015), but to our knowledge this is the first investigation of eukaryotic plankton diversity along the full salinity gradient using high-throughput sequencing. Compared to traditional microscopy based methods, high-throughput sequencing of taxonomic marker genes (metabarcoding) potentially offers a more comprehensive view of microbial diversity. While not yielding absolute cell counts, sequencing data presents integral information of microbial communities, and reveals trends in community changes. While morphological features in some cases can be used to classify plankton with high taxonomic resolution, this is not the case for the majority of species that are too small to allow classification by light microscopy. This is probably the reason why, with only 1 week's sampling, the present metabarcoding-based study revealed a microbial diversity on par with what has been recorded in the Baltic Sea with microscopy over a century (Hällfors, 2004; Telesh et al., 2009). In total we observed 1860 OTUs (548 broadly classified as phytoplankton and 774 as heterotrophs) which can be compared with 2075 known species of phytoplankton (Hällfors, 2004) and 1030 known species of micro-, meso-, and macrozooplankton (Telesh et al., 2009).

When performing metabarcoding, the taxonomic accuracy is limited primarily by the resolution of the marker gene, the coverage of the primers, and the quality and completeness of the reference database. Here we used the SSU rRNA gene, the most widely used taxonomic marker for bacterioplankton and protists (Amann et al., 1995; Pace, 1997). While it provides a lower taxonomic resolution than e.g., the internal transcribed spacer (ITS) region, its highly conserved regions allow the use of primers with domain-wide taxonomic range. Databases are also more complete than for other markers. We amplified and sequenced 450–600 bp of the hypervariable regions V3–V4 in bacteria and V4–V5 in eukaryotes, using in-house designed primer pairs that cover a large proportion of taxa in each domain (Hugerth et al., 2014a,b). Of the generated 18S OTUs, 31.8% could be classified to the genus level using our automatic classification scheme (see Methods), while 58.3% did not have sufficiently close matches (>98% identity) in the PR2 database (i.e., insufficient database completeness) and 9.8% matched multiple genera (i.e., insufficient taxonomic resolution of the marker). An example of the former condition is present for sequences classified to Amoebozoa, which displayed unusually low identity levels to reference sequences (Figure 2). Interestingly, the bacterial phylum Chlamydiae, that are symbionts on e.g., fish and amoeba in aquatic environments (Collingro et al., 2005; Stride et al., 2014) also displayed unusually low identity to reference sequences. Thus, the Baltic Sea appears to be inhabited by a set of poorly characterized Amoebozoa with poorly characterized bacterial symbionts. Examples of the latter were *E. Huxleyi* and *G. muellerae* (Bendif et al., 2015) that have 100% identical sequences in the V4–V5 region, as was also the case for species of Labyrinthula isolated from different hosts. Ideally, one would like to sequence both a part of the SSU rRNA gene and a more rapidly evolving marker, such as the ITS. With recently developed emulsion-based protocols, it will be possible to connect different markers from the same cell (Borgström et al., 2015; Spencer et al., 2016).

OTU relative abundances need to be carefully interpreted due to variations in the respective copy numbers of rRNA genes among phylogenetic groups, especially for eukaryotes (Prokopowich et al., 2003; Větrovský and Baldrian, 2013). Due to large cell volumes and vast genomes, dinoflagellates, diatoms, and ciliates in general possess more copies of the 18S rRNA gene than smaller flagellates, such as prasinophytes (Zhu et al., 2005; Godhe et al., 2008; Gong et al., 2013). Although, previous studies have shown a correlation between cell size (volume or length) and rRNA gene copy number (Godhe et al., 2008), copy numbers can also vary among species of the same genus. Thus, comparing the relative abundance among different taxonomic groups may be misleading. However, the variation in relative abundance of the same group across samples is still valid, allowing to test for correlations with variations in e.g., environmental parameters. Most taxonomic groups, at all taxonomic levels, displayed strikingly smooth abundance changes along the salinity gradient sampled (Figures 3–5, Supplementary Figure 6) in contrast to the spiky abundance distributions to be expected if the methodology had introduced substantial random noise. This was also true for metazoa (Figure 6), which is perhaps unexpected given that the low volumes of water sampled here (<1 l) are suboptimal for quantifying metazoa with their relatively low abundances in the water column. Systematic biases still likely exist, caused by e.g., primer mismatches and differences in rRNA gene copy numbers, but these should affect all samples equally.

Extracellular DNA released from dead (Kloos et al., 1994; Choi et al., 2004; Rice et al., 2007) or live (Böckelmann et al., 2006) cells may also lead to skewed estimates of abundance and diversity. Furthermore, metabarcoding detects all life forms that carry the marker gene, muddling the distinction between vegetative cells and other life stages. Some species of dinoflagellates, chrysophytes, cryptophytes, and haptophytes are known to form resting cysts after blooms (Lichtlé, 1980; Adam and Mahood, 1981; Jordan and Chamberlain, 1997; Lotter et al., 1998; Warns et al., 2012). One example is *Alexandrium*, a toxic genus of dinoflagellates (Teegarden and Cembella, 1996) that sporadically blooms in the Baltic (Witek, 2004; Kremp et al., 2009), for which we observed high abundance (9.1% of protist reads) in one of the Kattegat samples (Supplementary Figure 6B). Whether this reflects a bloom is hard to tell, since the sequences may be derived from cysts (Oshima et al., 1992). However, cysts are also toxic (Oshima et al., 1992), thus in some cases detecting both cysts and vegetative cells can be useful to track toxic outbreaks.

Similar to what was observed in previous studies (Herlemann et al., 2011; Dupont et al., 2014), bacterial community composition changed gradually along the salinity gradient, and difference in community composition (beta-diversity) was significantly correlated with difference in salinity. Here we showed for the first time a similar pattern for eukaryotic plankton composition using molecular data. The observed pattern can probably also be driven by a combination of other factors such as availability of micronutrients and composition of dissolved organic matter that covary with salinity across the gradient, and may be different or weaker during other seasons than summer, but it is well established that salinity difference *per se* is a major barrier for species to cross (Logares et al., 2009). A recent study shows that the bacterioplankton inhabiting the Baltic Proper are not locally adapted freshwater or marine populations, but are rather members of a global brackish metacommunity that most likely adapted to brackish conditions before the Baltic Sea was formed (Hugerth et al., 2015). Whether this also holds true for protists inhabiting this sea remains to be investigated. For endosymbiotic protists, isolated from the surrounding water and protected from osmotic stress, the distribution patterns are likely mainly determined by the salinity preference of the host.

The “Remane curve” (Remane, 1934) illustrates how diversity of freshwater, marine, and brackish specialist species vary along a salinity gradient. Total diversity is lowest in the horohaliculum (salinity 5–8) because few freshwater and marine species can tolerate this salinity level and relatively few true brackish specialists exist that occupy this niche. Remane based his “Artenminimum” (species-minimum) concept (Remane and Schlieper, 1971) on macrozoobenthos, but since then the generality of the pattern has been debated (Reid, 1961; McLusky, 1989; Attrill, 2002; Portillo et al., 2012). Recently, Telesh et al. suggested the opposite pattern for protists, with a species-maximum in the horohaliculum (Telesh et al., 2011b). The analysis was based on pooling species observations within salinity ranges from multiple studies, but has been questioned, since the intermediate salinity where the highest diversity was observed was also the most frequently sampled (Ptacnik et al., 2011; Telesh et al., 2011a), hence the pattern could stem from sampling biases. Here we took the opportunity to measure alpha-diversity with our metabarcoding dataset. This type of data has the potential to give an estimate of diversity that is not biased by morphological characters of the specimens, and the deep sequencing allows subsampling of each sample to the same depth, improving comparisons between samples (Lundin et al., 2012). Similar to what was previously observed (Herlemann et al., 2011), total bacterial alpha-diversity did not display any clear trends over the freshwater-marine continuum. Likewise, the total protist community (eukaryotic data excluding metazoa) displayed neither a species-minimum nor a species-maximum in the horohaliculum. When zooming in on specific eukaryotic groups, only one phyla, Ochrophyta, displayed significantly different diversity in the horohaliculum. Ochrophyta includes among other groups the diatoms, and since the sampling was conducted well after the spring bloom when diatoms are abundant in the Baltic Proper, the pattern may have been different if seasonal sampling had been conducted. Overall, there is considerable noise in the data, and the limited number of samples were collected during a single cruise in the summer, thus more data is obviously needed to derive stronger conclusions. However, if pronounced differences in alpha-diversity did exist between salinity regimes, this should have been visible. Telesh attributed the lack of an “Artenminimum” for protists to their fast evolutionary rate and hence ability to adapt to brackish conditions (as opposed to macroorganisms; Telesh et al., 2011b), which is also what was suggested for bacteria (Herlemann et al., 2011). However, our recent study showing that many of the bacterioplankton inhabiting the Baltic Sea are brackish specialists adapted to these conditions before the Baltic Sea was formed (Hugerth et al., 2015), and likely migrated from other brackish waters, opens the possibility that the same applies to some of the protist species. Future population genomic analysis may be able to reveal such patterns.

## Author contributions

YH, BK, and AA conceived and designed the study. BK performed sampling. YH performed molecular work. YH, AA analyzed the data. All authors wrote the paper, and read and approved the final version of the manuscript.

## Funding

This work was supported by BONUS BLUEPRINT project, supported by BONUS (Art 185), funded jointly by the EU and the Swedish Research Council FORMAS. It is also funded by the Swedish Research Council VR (grant 2011-5689) through a grant to AA. The work was in part supported by the JERICO project from the European Union Seventh Framework Programme (FP7/2007–2013) under grant agreement no. 262584, and through the Swedish Research Council VR through the Swedish Lifewatch project. YH was supported by a scholarship from the China Scholarship Council (CSC#201206950024).

### Conflict of interest statement

The authors declare that the research was conducted in the absence of any commercial or financial relationships that could be construed as a potential conflict of interest.

## References

[B1] Adam D. P. Mahood A. D. (1981). Chrysophyte cysts as potential environmental indicators. Geol. Soc. Am. Bull. 92, 839–844. 10.1130/0016-7606(1981)92<839:CCAPEI>2.0.CO;2

[B2] Altschul S. F. Madden T. L. Schäffer A. A. Zhang J. Zhang Z. Miller W.. (1997). Gapped BLAST and PSI-BLAST: a new generation of protein database search programs. Nucleic Acids Res. 25, 3389–3402. 10.1093/nar/25.17.3389 9254694 PMC146917

[B3] Amann R. I. Ludwig W. Schleifer K. H. (1995). Phylogenetic identification and in situ detection of individual microbial cells without cultivation. Microbiol. Rev. 59, 143–169. 10.1128/mr.59.1.143-169.1995 PMC239358 7535888

[B4] Amaral-Zettler L. A. McCliment E. A. Ducklow H. W. Huse S. M. (2009). A method for studying protistan diversity using massively parallel sequencing of V9 hypervariable regions of small-subunit ribosomal RNA genes. PLoS ONE 4:e6372. 10.1371/journal.pone.0006372 19633714 PMC2711349

[B5] Andersson A. F. Riemann L. Bertilsson S. (2010). Pyrosequencing reveals contrasting seasonal dynamics of taxa within Baltic Sea bacterioplankton communities. ISME J. 4, 171–181. 10.1038/ismej.2009.108 19829318

[B6] Attrill M. J. (2002). A testable linear model for diversity trends in estuaries. J. Anim. Ecol. 71, 262–269. 10.1046/j.1365-2656.2002.00593.x

[B7] Azam F. F. T. Fenchel T. Field J. G. Gray J. S. Meyer L. A. Thingstad F. (1983). The ecological role of water-column microbes in the Sea. Mar. Ecol. Prog. Ser. 10, 257–263. 10.3354/meps010257

[B8] Azam F. Malfatti F. (2007). Microbial structuring of marine ecosystems. Nat. Rev. Microbiol. 5, 782–791. 10.1038/nrmicro1747 17853906

[B9] Bass D. Chao E. E.-Y. Nikolaev S. Yabuki A. Ishida K.-I. Berney C.. (2009). Phylogeny of novel naked Filose and Reticulose Cercozoa: Granofilosea cl. n. and Proteomyxidea revised. Protist 160, 75–109. 10.1016/j.protis.2008.07.002 18952499

[B10] Behnke A. Barger K. J. Bunge J. Stoeck T. (2010). Spatio-temporal variations in protistan communities along an O2/H2S gradient in the anoxic Framvaren Fjord (Norway). FEMS Microbiol. Ecol. 72, 89–102. 10.1111/j.1574-6941.2010.00836.x 20163477

[B11] Beman J. M. Steele J. A. Fuhrman J. A. (2011). Co-occurrence patterns for abundant marine archaeal and bacterial lineages in the deep chlorophyll maximum of coastal California. ISME J. 5, 1077–1085. 10.1038/ismej.2010.204 21228895 PMC3146281

[B12] Bendif E. M. Probert I. Young J. R. von Dassow P. (2015). Morphological and phylogenetic characterization of new gephyrocapsa isolates suggests introgressive hybridization in the Emiliania/Gephyrocapsa complex (Haptophyta). Protist 166, 323–336. 10.1016/j.protis.2015.05.003 26037697

[B13] Bergen B. Herlemann D. P. R. Labrenz M. Jürgens K. (2014). Distribution of the verrucomicrobial clade Spartobacteria along a salinity gradient in the Baltic Sea. Environ. Microbiol. Rep. 6, 625–630. 10.1111/1758-2229.12178 25756116

[B14] Böckelmann U. Janke A. Kuhn R. Neu T. R. Wecke J. Lawrence J. R.. (2006). Bacterial extracellular DNA forming a defined network-like structure. FEMS Microbiol. Lett. 262, 31–38. 10.1111/j.1574-6968.2006.00361.x 16907736

[B15] Borgström E. Redin D. Lundin S. Berglund E. Andersson A. F. Ahmadian A. (2015). Phasing of single DNA molecules by massively parallel barcoding. Nat. Commun. 6, 7173. 10.1038/ncomms8173 26055759 PMC4468844

[B16] Carlson C. A. Morris R. Parsons R. Treusch A. H. Giovannoni S. J. Vergin K. (2009). Seasonal dynamics of SAR11 populations in the euphotic and mesopelagic zones of the northwestern Sargasso Sea. ISME J. 3, 283–295. 10.1038/ismej.2008.117 19052630

[B17] Carstensen J. Andersen J. H. Gustafsson B. G. Conley D. J. (2014). Deoxygenation of the Baltic Sea during the last century. Proc. Natl. Acad. Sci. U.S.A. 111, 5628–5633. 10.1073/pnas.1323156111 24706804 PMC3992700

[B18] Casini M. Hjelm J. Molinero J.-C. Lövgren J. Cardinale M. Bartolino V.. (2009). Trophic cascades promote threshold-like shifts in pelagic marine ecosystems. Proc. Natl. Acad. Sci. U.S.A. 106, 197–202. 10.1073/pnas.0806649105 19109431 PMC2629246

[B19] Cavalier-Smith T. (1998). A revised six-kingdom system of life. Biol. Rev. Camb. Philos. Soc. 73, 203–266. 10.1017/S0006323198005167 9809012

[B20] Choi J.-J. Reich C. F. III. Pisetsky D. S. (2004). Release of DNA from dead and dying lymphocyte and monocyte cell lines *in vitro*. Scand. J. Immunol. 60, 159–166. 10.1111/j.0300-9475.2004.01470.x 15238085

[B21] Cole J. R. Wang Q. Fish J. A. Chai B. McGarrell D. M. Sun Y.. (2014). Ribosomal Database Project: data and tools for high throughput rRNA analysis. Nucleic Acids Res. 42, D633–D642. 10.1093/nar/gkt1244 24288368 PMC3965039

[B22] Collingro A. Toenshoff E. R. Taylor M. W. Fritsche T. R. Wagner M. Horn M. (2005). "Candidatus Protochlamydia amoebophila", an endosymbiont of Acanthamoeba spp. Int. J. Syst. Evol. Microbiol. 55, 1863–1866. 10.1099/ijs.0.63572-0 16166679

[B23] Decelle J. Romac S. Stern R. F. Bendif E. M. Zingone A. Audic S.. (2015). PhytoREF: a reference database of the plastidial 16S rRNA gene of photosynthetic eukaryotes with curated taxonomy. Mol. Ecol. Resour. 15, 1435–1445. 10.1111/1755-0998.12401 25740460

[B24] Demir-Hilton E. Sudek S. Cuvelier M. L. Gentemann C. L. Zehr J. P. Worden A. Z. (2011). Global distribution patterns of distinct clades of the photosynthetic picoeukaryote Ostreococcus. ISME J. 5, 1095–1107. 10.1038/ismej.2010.209 21289652 PMC3146286

[B25] de Vargas C. Audic S. Henry N. Decelle J. Mah,é F. Logares R.. (2015). Ocean plankton. Eukaryotic plankton diversity in the sunlit ocean. Science 348:1261605. 10.1126/science.1261605 25999516

[B26] Di Rienzi S. C. Sharon I. Wrighton K. C. Koren O. Hug L. A. Thomas B. C.. (2013). The human gut and groundwater harbor non-photosynthetic bacteria belonging to a new candidate phylum sibling to Cyanobacteria. Elife 2:e01102. 10.7554/elife.01102 24137540 PMC3787301

[B27] Dupont C. L. Larsson J. Yooseph S. Ininbergs K. Goll J. Asplund-Samuelsson J.. (2014). Functional tradeoffs underpin salinity-driven divergence in microbial community composition. PLoS ONE 9:e89549. 10.1371/journal.pone.0089549 24586863 PMC3937345

[B28] Edgar R. C. (2010). Search and clustering orders of magnitude faster than BLAST. Bioinformatics 26, 2460–2461. 10.1093/bioinformatics/btq461 20709691

[B29] Eiler A. Drakare S. Bertilsson S. Pernthaler J. Peura S. Rofner C.. (2013). Unveiling distribution patterns of freshwater phytoplankton by a next generation sequencing based approach. PLoS ONE 8:e53516. 10.1371/journal.pone.0053516 23349714 PMC3551911

[B30] Falkowski P. G. Fenchel T. Delong E. F. (2008). The microbial engines that drive Earth's biogeochemical cycles. Science 320, 1034–1039. 10.1126/science.1153213 18497287

[B31] Godhe A. Asplund M. E. Härnström K. Saravanan V. Tyagi A. Karunasagar I. (2008). Quantification of diatom and dinoflagellate biomasses in coastal marine seawater samples by real-time PCR. Appl. Environ. Microbiol. 74, 7174–7182. 10.1128/AEM.01298-08 18849462 PMC2592920

[B32] Gong J. Dong J. Liu X. Massana R. (2013). Extremely high copy numbers and polymorphisms of the rDNA operon estimated from single cell analysis of oligotrich and peritrich ciliates. Protist 164, 369–379. 10.1016/j.protis.2012.11.006 23352655

[B33] Gorokhova E. Hajdu S. Larsson U. (2014). Responses of phyto- and zooplankton communities to Prymnesium polylepis (Prymnesiales) bloom in the Baltic Sea. PLoS ONE 9:e112985. 10.1371/journal.pone.0112985 25393031 PMC4231118

[B34] Grote J. Thrash J. C. Huggett M. J. Landry Z. C. Carini P. Giovannoni S. J.. (2012). Streamlining and core genome conservation among highly divergent members of the SAR11 clade. MBio 3:e00252-12. 10.1128/mBio.00252-12 22991429 PMC3448164

[B35] Guillou L. Bachar D. Audic S. Bass D. Berney C. Bittner L.. (2013). The Protist Ribosomal Reference database (PR2): a catalog of unicellular eukaryote small sub-unit rRNA sequences with curated taxonomy. Nucleic Acids Res. 41, D597–D604. 10.1093/nar/gks1160 23193267 PMC3531120

[B36] Guillou L. Viprey M. Chambouvet A. Welsh R. M. Kirkham A. R. Massana R.. (2008). Widespread occurrence and genetic diversity of marine parasitoids belonging to Syndiniales (Alveolata). Environ. Microbiol. 10, 3349–3365. 10.1111/j.1462-2920.2008.01731.x 18771501

[B37] Hällfors G. (2004). Checklist of Baltic Sea Phytoplankton Species. Helsinki Commission; Baltic Marine Environment protection Commission. Available online at: http://helcom.fi/Lists/Publications/BSEP95.pdf

[B38] HELCOM (2015). Manual for Marine Monitoring in the COMBINE Programme of HELCOM. ICES,HELCOM. Available online at: http://www.helcom.fi/Documents/Action%20areas/Monitoring%20and%20assessment/Manuals%20and%20Guidelines/Manual%20for%20Marine%20Monitoring%20in%20the%20COMBINE%20Programme%20of%20HELCOM.pdf

[B39] Herlemann D. P. Labrenz M. Jürgens K. Bertilsson S. Waniek J. J. Andersson A. F. (2011). Transitions in bacterial communities along the 2000 km salinity gradient of the Baltic Sea. ISME J. 5, 1571–1579. 10.1038/ismej.2011.41 21472016 PMC3176514

[B40] Herlemann D. P. R. Lundin D. Labrenz M. Jürgens K. Zheng Z. Aspeborg H.. (2013). Metagenomic de novo assembly of an aquatic representative of the verrucomicrobial class Spartobacteria. MBio 4, e00569–e00512. 10.1128/mBio.00569-12 23716574 PMC3663571

[B41] Hugerth L. W. Larsson J. Alneberg J. Lindh M. V. Legrand C. Pinhassi J.. (2015). Metagenome-assembled genomes uncover a global brackish microbiome. Genome Biol. 16, 279. 10.1186/s13059-015-0834-7 26667648 PMC4699468

[B42] Hugerth L. W. Muller E. E. L. Hu Y. O. O. Lebrun L. A. M. Roume H. Lundin D.. (2014a). Systematic design of 18S rRNA gene primers for determining eukaryotic diversity in microbial consortia. PLoS ONE 9:e95567. 10.1371/journal.pone.0095567 24755918 PMC3995771

[B43] Hugerth L. W. Wefer H. A. Lundin S. Jakobsson H. E. Lindberg M. Rodin S.. (2014b). DegePrime, a program for degenerate primer design for broad-taxonomic-range PCR in microbial ecology studies. Appl. Environ. Microbiol. 80, 5116–5123. 10.1128/AEM.01403-14 24928874 PMC4135748

[B44] Johannesson K. André C. (2006). Life on the margin: genetic isolation and diversity loss in a peripheral marine ecosystem, the Baltic Sea. Mol. Ecol. 15, 2013–2029. 10.1111/j.1365-294X.2006.02919.x 16780421

[B45] Johansson M. Gorokhova E. Larsson U. (2004). Annual variability in ciliate community structure, potential prey and predators in the open northern Baltic Sea proper. J. Plankton Res. 26, 67–80. 10.1093/plankt/fbg115

[B46] Jordan R. W. Chamberlain A. H. L. (1997). Biodiversity among haptophyte algae. Biodivers. Conserv. 6, 131–152. 10.1023/A:1018383817777

[B47] Karlson B. Andersson L. S. Kaitala S. Kronsell J. Mohlin M. Seppäla J. (in press). A comparison of Ferrybox data vs. monitoring data from research vessels for near surface waters of the Baltic Sea the Kattegat. J. Mar. Syst.

[B48] Keeling P. J. (2007). Ostreococcus tauri: seeing through the genes to the genome. Trends Genet. 23, 151–154. 10.1016/j.tig.2007.02.008 17331615

[B49] Kloos D. U. Strätz M. Güttler A. Steffan R. J. Timmis K. N. (1994). Inducible cell lysis system for the study of natural transformation and environmental fate of DNA released by cell death. J. Bacteriol. 176, 7352–7361. 7961508 10.1128/jb.176.23.7352-7361.1994 PMC197125

[B50] Kremp A. Lindholm T. Dreßler N. Erler K. Gerdts G. Eirtovaara S. (2009). Bloom forming Alexandrium ostenfeldii (Dinophyceae) in shallow waters of the Åland Archipelago, Northern Baltic Sea. Harmful Algae 8, 318–328. 10.1016/j.hal.2008.07.004

[B51] Larsson J. Celepli N. Ininbergs K. Dupont C. L. Yooseph S. Bergman B.. (2014). Picocyanobacteria containing a novel pigment gene cluster dominate the brackish water Baltic Sea. ISME J. 8, 1892–1903. 10.1038/ismej.2014.35 24621524 PMC4139726

[B52] Lemieux C. Otis C. Turmel M. (2014). Chloroplast phylogenomic analysis resolves deep-level relationships within the green algal class Trebouxiophyceae. BMC Evol. Biol. 14:211. 10.1186/s12862-014-0211-2 25270575 PMC4189289

[B53] Lichtlé C. (1980). Effects of nitrogen deficiency and light of high intensity on Cryptomonas rufescens (Cryptophyceae). Protoplasma 102, 11–19. 10.1007/BF01276944

[B54] Lin Y.-C. Campbell T. Chung C.-C. Gong G.-C. Chiang K.-P. Worden A. Z. (2012). Distribution patterns and phylogeny of marine stramenopiles in the north pacific ocean. Appl. Environ. Microbiol. 78, 3387–3399. 10.1128/AEM.06952-11 22344659 PMC3346489

[B55] Lindh M. V. Sjöstedt J. Andersson A. F. Baltar F. Hugerth L. W. Lundin D.. (2015). Disentangling seasonal bacterioplankton population dynamics by high-frequency sampling. Environ. Microbiol. 17, 2459–2476. 10.1111/1462-2920.12720 25403576

[B56] Logares R. Bråte J. Bertilsson S. Clasen J. L. Shalchian-Tabrizi K. Rengefors K. (2009). Infrequent marine-freshwater transitions in the microbial world. Trends Microbiol. 17, 414–422. 10.1016/j.tim.2009.05.010 19726194

[B57] Lotter A. F. Birks H. J. B. Hofmann W. Marchetto A. (1998). Modern diatom, cladocera, chironomid, and chrysophyte cyst assemblages as quantitative indicators for the reconstruction of past environmental conditions in the Alps. II. Nutrients. J. Paleolimnol. 19, 443–463. 10.1023/A:1007994206432

[B58] Lovejoy C. Massana R. Pedrós-Alió C. (2006). Diversity and distribution of marine microbial eukaryotes in the Arctic Ocean and adjacent seas. Appl. Environ. Microbiol. 72, 3085–3095. 10.1128/AEM.72.5.3085-3095.2006 16672445 PMC1472370

[B59] Lundin D. Severin I. Logue J. B. Ostman O. Andersson A. F. Lindström E. S. (2012). Which sequencing depth is sufficient to describe patterns in bacterial α- and β-diversity? Environ. Microbiol. Rep. 4, 367–372. 10.1111/j.1758-2229.2012.00345.x 23760801

[B60] Lundin S. Stranneheim H. Pettersson E. Klevebring D. Lundeberg J. (2010). Increased throughput by parallelization of library preparation for massive sequencing. PLoS ONE 5:e10029. 10.1371/journal.pone.0010029 20386591 PMC2850305

[B61] Massana R. Castresana J. Balagué V. Guillou L. Romari K. Groisillier A.. (2004). Phylogenetic and ecological analysis of novel marine stramenopiles. Appl. Environ. Microbiol. 70, 3528–3534. 10.1128/AEM.70.6.3528-3534.2004 15184153 PMC427732

[B62] Massana R. del Campo J. Sieracki M. E. Audic S. Logares R. (2014). Exploring the uncultured microeukaryote majority in the oceans: reevaluation of ribogroups within stramenopiles. ISME J. 8, 854–866. 10.1038/ismej.2013.204 24196325 PMC3960543

[B63] Massana R. Terrado R. Forn I. Lovejoy C. Pedrós-Ali,ó C. (2006). Distribution and abundance of uncultured heterotrophic flagellates in the world oceans. Environ. Microbiol. 8, 1515–1522. 10.1111/j.1462-2920.2006.01042.x 16913912

[B64] McLusky D. S. (1989). The Estuarine Ecosystem. Glasgow: Blackie academic & professional.

[B65] Mironova E. I. Telesh I. V. Skarlato S. O. (2009). Planktonic ciliates of the Baltic Sea (a review). Inland Water Biol. 2, 13–24. 10.1134/S1995082909010039

[B66] Not F. del Campo J. Balagué V. de Vargas C. Massana R. (2009). New insights into the diversity of marine picoeukaryotes. PLoS ONE 4:e7143. 10.1371/journal.pone.0007143 19787059 PMC2747013

[B67] Not F. Valentin K. Romari K. Lovejoy C. Massana R. Töbe K.. (2007). Picobiliphytes: a marine picoplanktonic algal group with unknown affinities to other eukaryotes. Science 315, 253–255. 10.1126/science.1136264 17218530

[B68] Ojaveer H. Jaanus A. Mackenzie B. R. Martin G. Olenin S. Radziejewska T.. (2010). Status of biodiversity in the Baltic Sea. PLoS ONE 5:e12467. 10.1371/journal.pone.0012467 20824189 PMC2931693

[B69] Olenina I. Edler L. Andersson A. Wasmund N. Busch S. Göbel J. (2006). Biovolumes and Size-Classes of Phytoplankton in the Baltic Sea. Helsinki Commission; Baltic Marine Environment Protection Commission. Available online at: http://helcom.fi/Lists/Publications/BSEP106.pdf

[B70] Oshima Y. Bolch C. J. Hallegraeff G. M. (1992). Toxin composition of resting cysts of Alexandrium tamarense (Dinophyceae). Toxicon 30, 1539–1544. 10.1016/0041-0101(92)90025-Z 1488762

[B71] Pace N. R. (1997). A molecular view of microbial diversity and the biosphere. Science 276, 734–740. 10.1126/science.276.5313.734 9115194

[B72] Pearson W. R. Wood T. Zhang Z. Miller W. (1997). Comparison of DNA sequences with protein sequences. Genomics 46, 24–36. 10.1006/geno.1997.4995 9403055

[B73] Piwosz K. Pernthaler J. (2010). Seasonal population dynamics and trophic role of planktonic nanoflagellates in coastal surface waters of the Southern Baltic Sea. Environ. Microbiol. 12, 364–377. 10.1111/j.1462-2920.2009.02074.x 19799618

[B74] Portillo M. C. Anderson S. P. Fierer N. (2012). Temporal variability in the diversity and composition of stream bacterioplankton communities. Environ. Microbiol. 14, 2417–2428. 10.1111/j.1462-2920.2012.02785.x 22626459

[B75] Prokopowich C. D. Gregory T. R. Crease T. J. (2003). The correlation between rDNA copy number and genome size in eukaryotes. Genome 46, 48–50. 10.1139/g02-103 12669795

[B76] Ptacnik R. Olli K. Lehtinen S. Tamminen T. Andersen T. (2011). Does plankton diversity peak at intermediate salinities? Comment on Telesh et al. (2011). Mar. Ecol. Prog. Ser. 432, 291–292. 10.3354/meps09216

[B77] Quast C. Pruesse E. Yilmaz P. Gerken J. Schweer T. Yarza P.. (2013). The SILVA ribosomal RNA gene database project: improved data processing and web-based tools. Nucleic Acids Res. 41, D590–D596. 10.1093/nar/gks1219 23193283 PMC3531112

[B78] Reid G. K. (1961). Ecology of Inland Waters and Estuaries. Available online at: http://agris.fao.org/agris-search/search.do?recordID=US201300423127

[B79] Remane A. (1934). Die brackwasserfauna. Verh. Dtsch. Zool. Ges. 36, 34–74. 27109087

[B80] Remane A. Schlieper C. (1971). Biology of brackish water. Stuttgart: Schweizerbart.

[B81] Rice K. C. Mann E. E. Endres J. L. Weiss E. C. Cassat J. E. Smeltzer M. S.. (2007). The cidA murein hydrolase regulator contributes to DNA release and biofilm development in Staphylococcus aureus. Proc. Natl. Acad. Sci. U.S.A. 104, 8113–8118. 10.1073/pnas.0610226104 17452642 PMC1876580

[B82] Samuelsson K. Berglund J. Andersson A. (2006). Factors structuring the heterotrophic flagellate and ciliate community along a brackish water primary production gradient. J. Plankton Res. 28, 345–359. 10.1093/plankt/fbi118

[B83] Seenivasan R. Sausen N. Medlin L. K. Melkonian M. (2013). Picomonas judraskeda gen. et sp. nov., the first identified member of the Picozoa phylum nov., a widespread group of picoeukaryotes, formerly known as picobiliphytes. PLoS ONE 8:e59565. 10.1371/journal.pone.0059565 23555709 PMC3608682

[B84] Seppälä J. Ylöstalo P. Kaitala S. Hällfors S. Raateoja M. Maunula P. (2007). Ship-of-opportunity based phycocyanin fluorescence monitoring of the filamentous cyanobacteria bloom dynamics in the Baltic Sea. Estuar. Coast. Shelf Sci. 73, 489–500. 10.1016/j.ecss.2007.02.015

[B85] Sjöqvist C. O. Lindholm T. J. (2011). Natural co-occurrence of Dinophysis acuminata (Dinoflagellata) and Mesodinium rubrum (Ciliophora) in thin layers in a coastal inlet. J. Eukaryot. Microbiol. 58, 365–372. 10.1111/j.1550-7408.2011.00559.x 21569163

[B86] Sogin M. L. Morrison H. G. Huber J. A. Welch D. M. Huse S. M. Neal P. R.. (2006). Microbial diversity in the deep sea and the underexplored “rare biosphere. Proc. Natl. Acad. Sci. U.S.A. 103, 12115–12120. 10.1073/pnas.0605127103 16880384 PMC1524930

[B87] Spencer S. J. Tamminen M. V. Preheim S. P. Guo M. T. Briggs A. W. Brito I. L.. (2016). Massively parallel sequencing of single cells by epicPCR links functional genes with phylogenetic markers. ISME J. 10, 427–436. 10.1038/ismej.2015.124 26394010 PMC4737934

[B88] Stal L. J. Albertano P. Bergman B. Bröckel K. von Gallon, J. R. Hayes P. K. (2003). BASIC: Baltic Sea cyanobacteria. An investigation of the structure and dynamics of water blooms of cyanobacteria in the Baltic Sea—responses to a changing environment. Cont. Shelf Res. 23, 1695–1714. 10.1016/j.csr.2003.06.001

[B89] Stride M. C. Polkinghorne A. Nowak B. F. (2014). Chlamydial infections of fish: diverse pathogens and emerging causes of disease in aquaculture species. Vet. Microbiol. 170, 19–27. 10.1016/j.vetmic.2014.01.022 24560593

[B90] Subirana L. Péquin B. Michely S. Escande M.-L. Meilland J. Derelle E.. (2013). Morphology, genome plasticity, and phylogeny in the genus Ostreococcus reveal a cryptic species, *O. mediterraneus* sp. nov. (Mamiellales, Mamiellophyceae). Protist 164, 643–659. 10.1016/j.protis.2013.06.002 23892412

[B91] Teegarden G. J. Cembella A. D. (1996). Grazing of toxic dinoflagellates, Alexandrium spp., by adult copepods of coastal Maine: implications for the fate of paralytic shellfish toxins in marine food webs. J. Exp. Mar. Bio. Ecol. 196, 145–176. 10.1016/0022-0981(95)00128-X

[B92] Telesh I. Postel L. Heerkloss R. Mironova E. Skarlato S. (2009). Zooplankton of the Open Baltic Sea: Extended Atlas. Leibniz Institute. Available online at: http://www.io-warnemuende.de/tl_files/forschung/meereswissenschaftliche-berichte/mebe76_2009-zooplankton-extended-atlas.pdf

[B93] Telesh I. Schubert H. Skarlato S. (2013). Life in the salinity gradient: discovering mechanisms behind a new biodiversity pattern. Estuar. Coast. Shelf Sci. 135, 317–327. 10.1016/j.ecss.2013.10.013

[B94] Telesh I. V. Schubert H. Skarlato S. O. (2011a). Protistan diversity does peak in the horohalinicum of the Baltic Sea: reply to Ptacnik et al. (2011). Mar. Ecol. Prog. Ser. 432, 293–297. 10.3354/meps09217

[B95] Telesh I. V. Schubert H. Skarlato S. O. (2011b). Revisiting Remane's concept: evidence for high plankton diversity and a protistan species maximum in the horohalinicum of the Baltic Sea. Mar. Ecol. Prog. Ser. 421, 1–11. 10.3354/meps08928

[B96] Thaler M. Lovejoy C. (2013). Environmental selection of marine stramenopile clades in the Arctic Ocean and coastal waters. Polar Biol. 37, 347–357. 10.1007/s00300-013-1435-0

[B97] Thomsen H. A. Oates K. (1978). Balaniger balticus gen. et sp. nov.(Prymnesiophyceae) from Danish coastal waters. J. Mar. Biol. Assoc. U.K. 58, 773–779. 10.1017/S0025315400041424

[B98] Tyrrell T. Schneider B. Charalampopoulou A. Riebesell U. (2008). Coccolithophores and calcite saturation state in the Baltic and Black Seas. Biogeosciences 5, 485–494. 10.5194/bg-5-485-2008

[B99] Větrovský T. Baldrian P. (2013). The variability of the 16S rRNA gene in bacterial genomes and its consequences for bacterial community analyses. PLoS ONE 8:e57923. 10.1371/journal.pone.0057923 23460914 PMC3583900

[B100] Warns A. Hense I. Kremp A. (2012). Modelling the life cycle of dinoflagellates: a case study with *Biecheleria baltica*. J. Plankton Res. 35, 379–392. 10.1093/plankt/fbs095

[B101] Weber F. del Campo J. Wylezich C. Massana R. Jürgens K. (2012). Unveiling trophic functions of uncultured protist taxa by incubation experiments in the brackish Baltic Sea. PLoS ONE 7:e41970. 10.1371/journal.pone.0041970 22860041 PMC3408427

[B102] Witek B. (2004). Blooms of potentially toxic dinoflagellates Prorocentrum minimum (Pavillard) and Schiller and Alexandrium ostenfeldii (Paulsen) Balech and Tangen in the Gulf of Gdá nsk, in Ogó lnopolska Konferencja Naukowa Zakwity Wody-Monitoring i Kontrola Zagrozen (Gdynia).

[B103] Zhu F. Massana R. Not F. Marie D. Vaulot D. (2005). Mapping of picoeucaryotes in marine ecosystems with quantitative PCR of the 18S rRNA gene. FEMS Microbiol. Ecol. 52, 79–92. 10.1016/j.femsec.2004.10.006 16329895

