# Supplementary material for: Diversity of Pico- to Mesoplankton along the 2000 km Salinity Gradient of the Baltic Sea
Source: Front Microbiol. 2016 May 12;7:679. doi: 10.3389/fmicb.2016.00679 (PMC4864665; doi:10.3389/fmicb.2016.00679)
Supplement: Supplementary file 1 [file Image1.PDF]

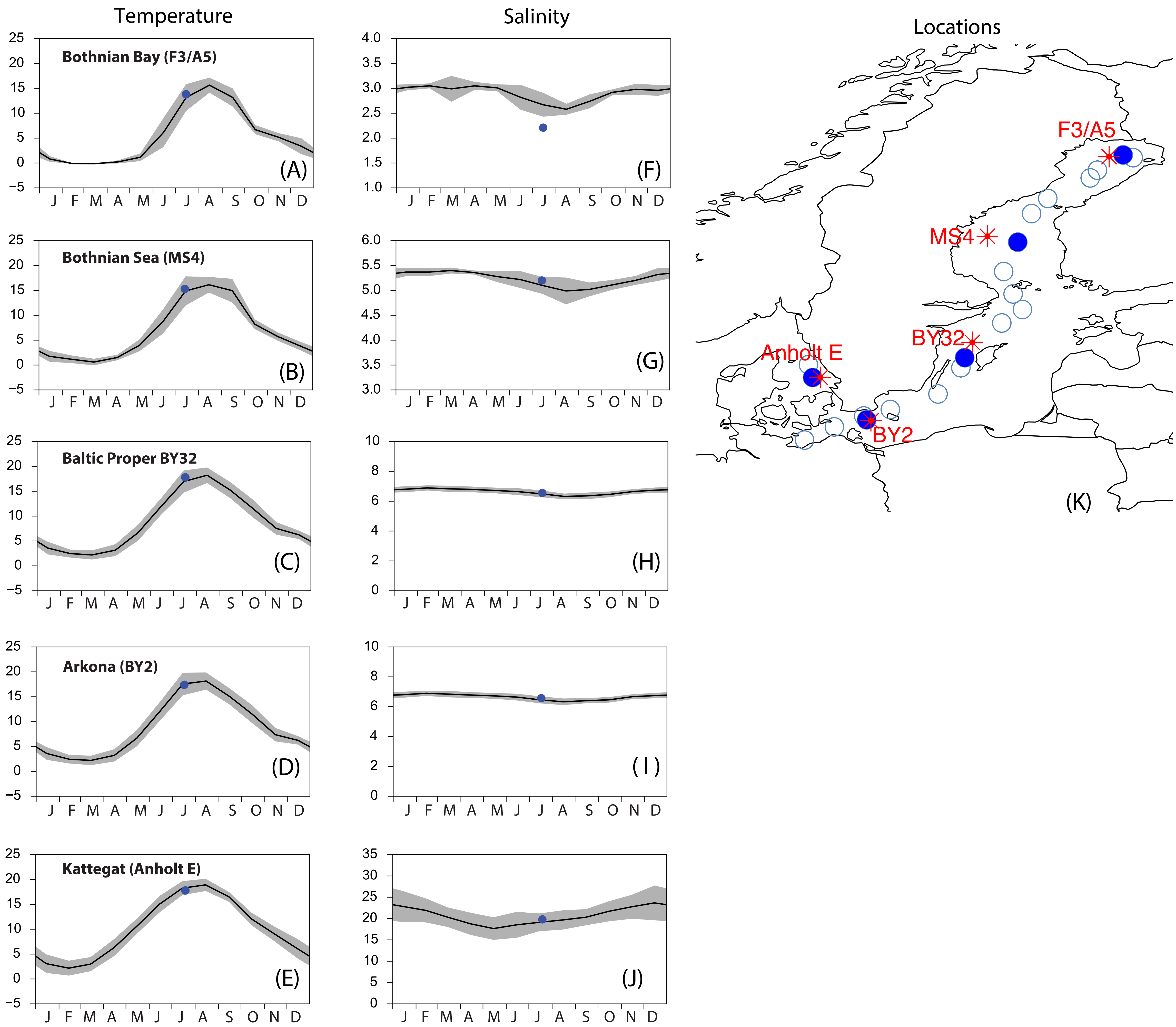

Supplementary figure 1. Annual variation in temperature and salinity in the different wter bodies sampled. (A - J) Monthly means (black lines) and standard deviations (grey shadings) of temperature (A - E) and salinity (F - J) for the period 2000 - 2015 for stations in the Swedish national marine monitoring program (http://sharkweb.smhi.se). Blue circles indicate data from the nearest stations in this study. **(K)** Map showing monitoring program stations (red stars), stations in this study (blue circles), and stations in this study included in panels A - J (filled blue circles). Note that the range of the y-axis for salinity differs between the plots F -J .
