# Supplementary material for: Diversity of Pico- to Mesoplankton along the 2000 km Salinity Gradient of the Baltic Sea
Source: Front Microbiol. 2016 May 12;7:679. doi: 10.3389/fmicb.2016.00679 (PMC4864665; doi:10.3389/fmicb.2016.00679)
Supplement: Supplementary file 2 [file Image2.PDF]

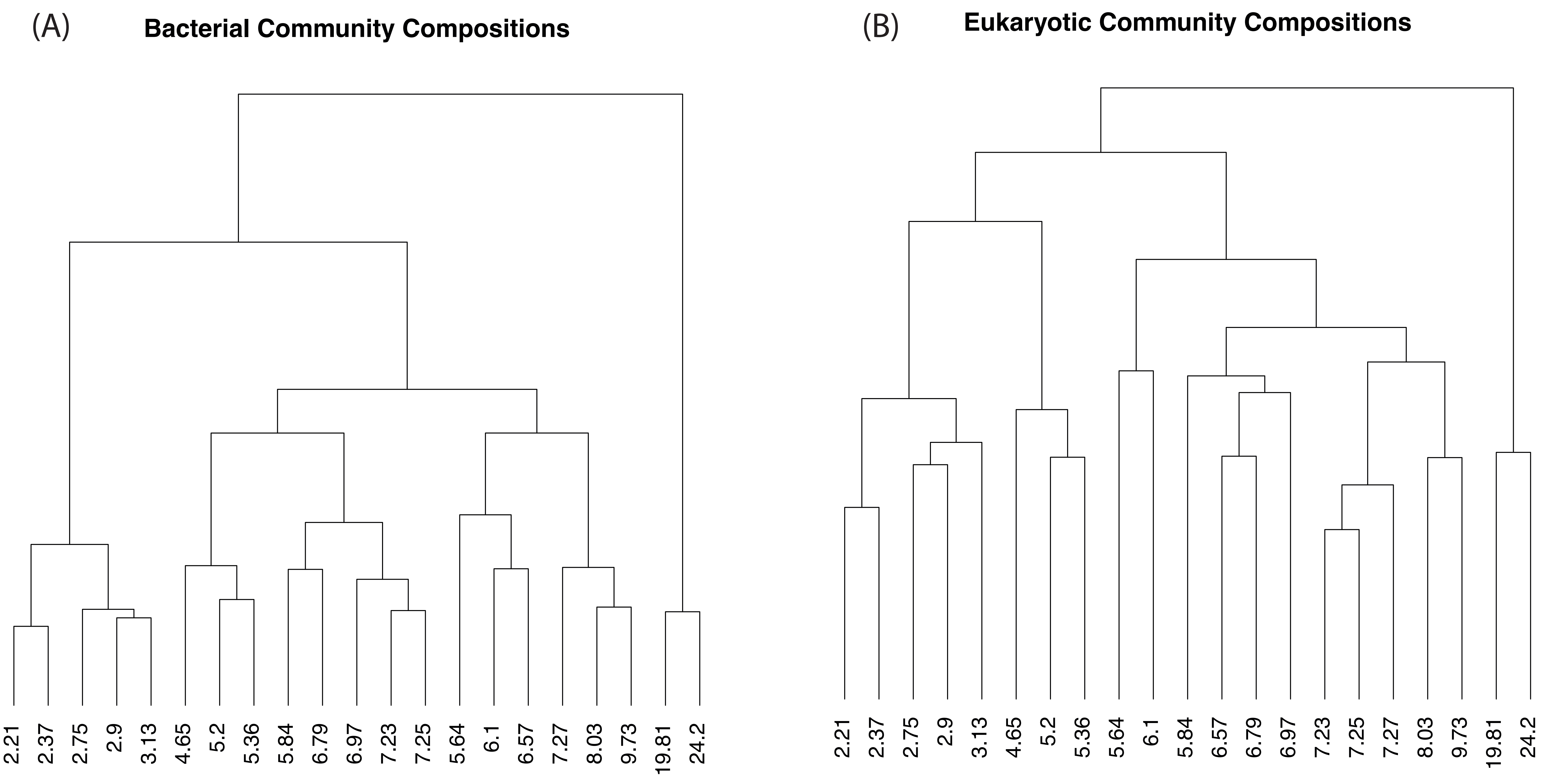

Supplementary figure 2. Hierarchical clustering of samples based on beta-diversity calculated using Spearman correlation of OTU frequencies. (A) Bacterial communities. **(B)** Eukaryotic communities.
