# Supplementary material for: Diversity of Pico- to Mesoplankton along the 2000 km Salinity Gradient of the Baltic Sea
Source: Front Microbiol. 2016 May 12;7:679. doi: 10.3389/fmicb.2016.00679 (PMC4864665; doi:10.3389/fmicb.2016.00679)
Supplement: Supplementary file 3 [file Image3.PDF]

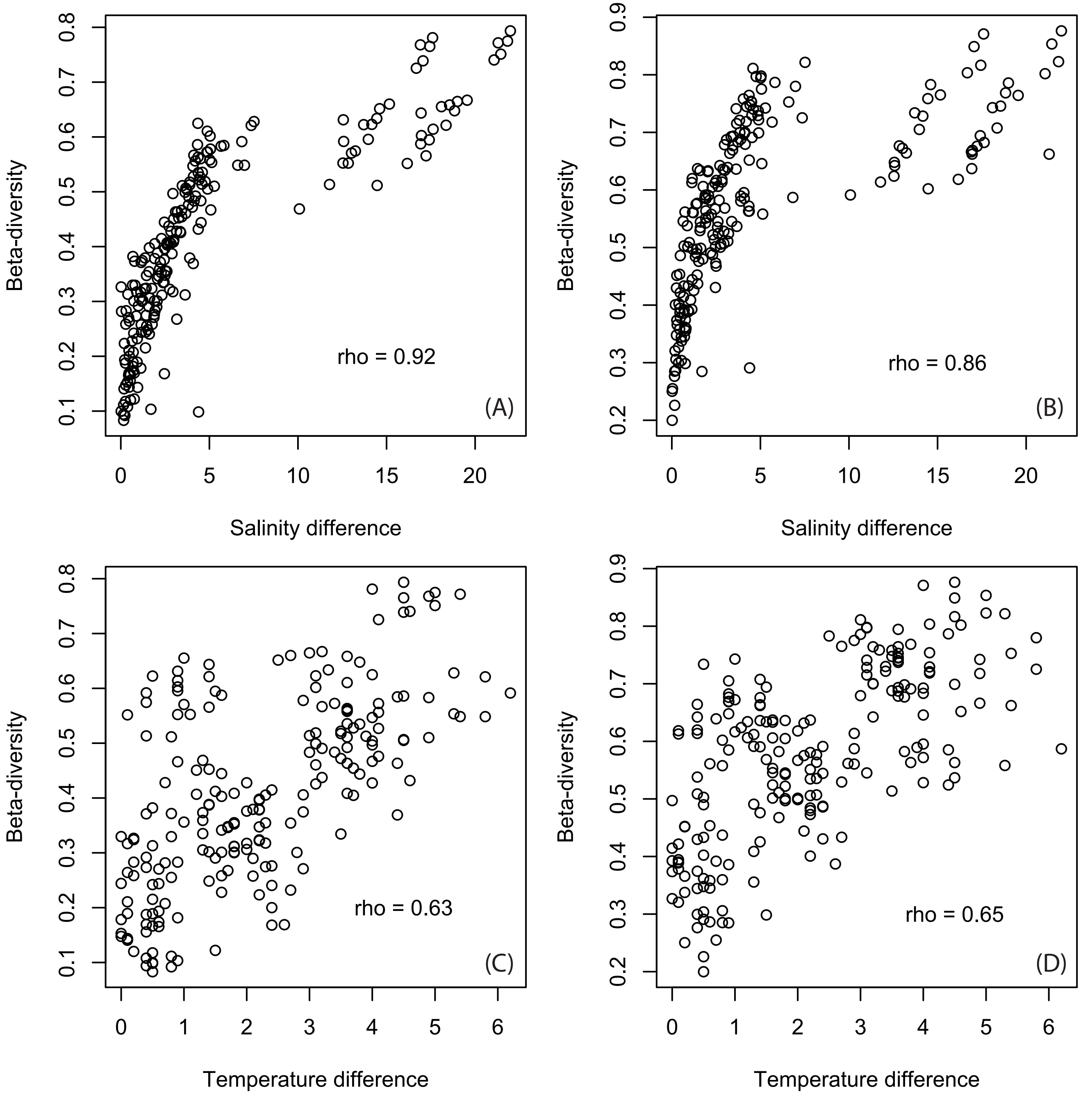

Supplementary figure 3. Correspondence between beta-diversity (community difference) and (absolute) difference in salinity and temperature. (A,C) Bacterial communities. **(B,D)** Eukaryotic communities. Each circle is one pair of samples. The Spearman correlation coefficient (rho) is indicated in each plot.
