# Supplementary material for: Diversity of Pico- to Mesoplankton along the 2000 km Salinity Gradient of the Baltic Sea
Source: Front Microbiol. 2016 May 12;7:679. doi: 10.3389/fmicb.2016.00679 (PMC4864665; doi:10.3389/fmicb.2016.00679)
Supplement: Supplementary file 4 [file Image4.PDF]

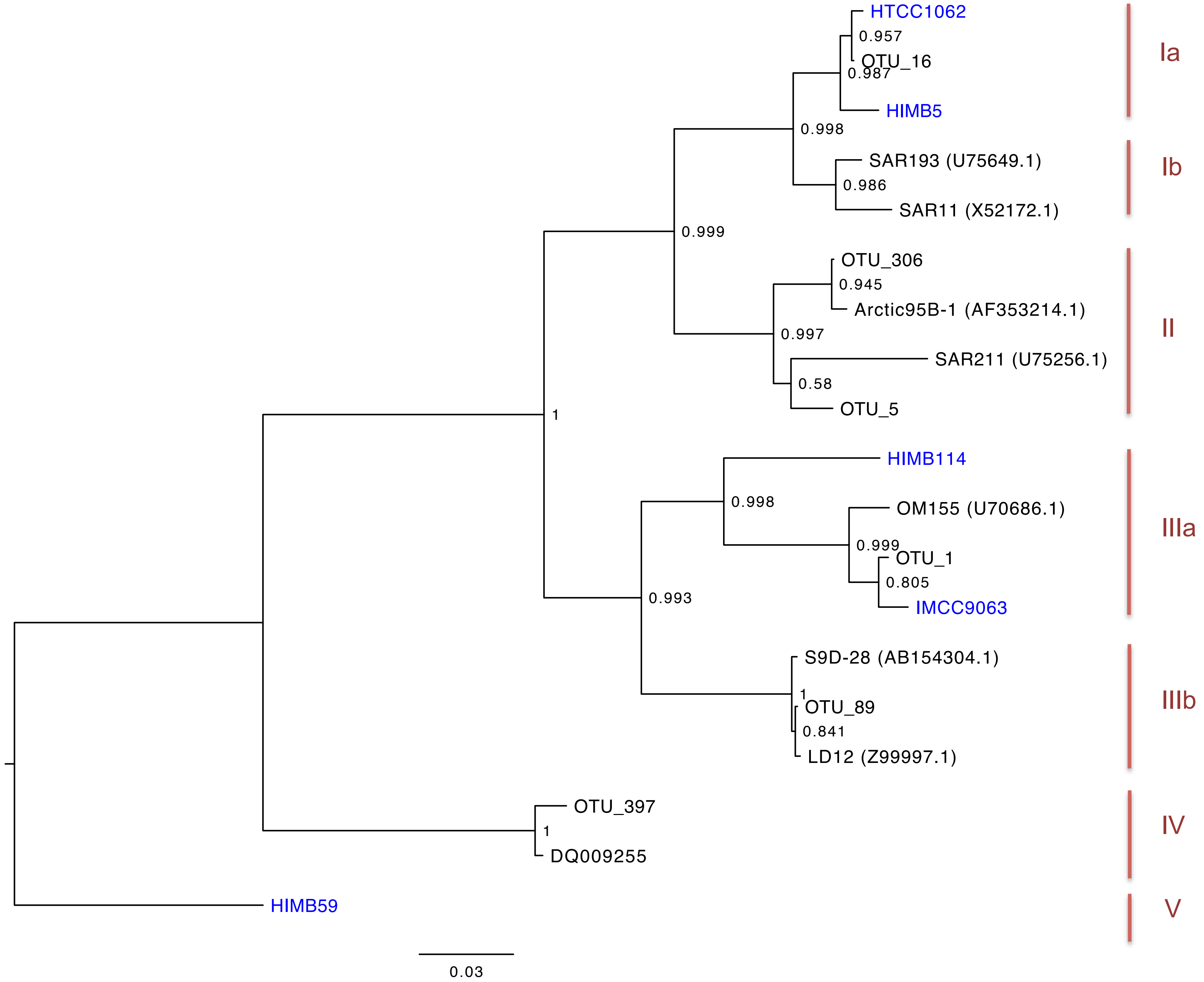

Supplementary figure 4. Maximum-likelihood phylogenetic tree of SAR11 OTU sequences and reference sequences. Reference sequences and subcluster annotations are based on Figure 1 in Grote et al (Grote 2012), supplemented with IMCC9063. The tree was rooted using HIMB59 as outgroup. Numbers at nodes indicate the reliability of the corresponding split in the tree using the Shimodaira-Hasegawa test (ranging from 0 to 1). Blue-colored references represent genome-sequenced isolates. Pairwise identities between OTU sequences and their nearest neighbors in the tree were as follows: OTU_16 - HTCC1062: 100%; OTU_306 - Arctic95B-1: 99.7%; OTU_1 - IMCC9063: 99.5%; OTU_89 - LD12: 100%; OTU_397 - DQ009255: 99%.
