# Supplementary material for: Diversity of Pico- to Mesoplankton along the 2000 km Salinity Gradient of the Baltic Sea
Source: Front Microbiol. 2016 May 12;7:679. doi: 10.3389/fmicb.2016.00679 (PMC4864665; doi:10.3389/fmicb.2016.00679)
Supplement: Supplementary file 5 [file Image5.PDF]

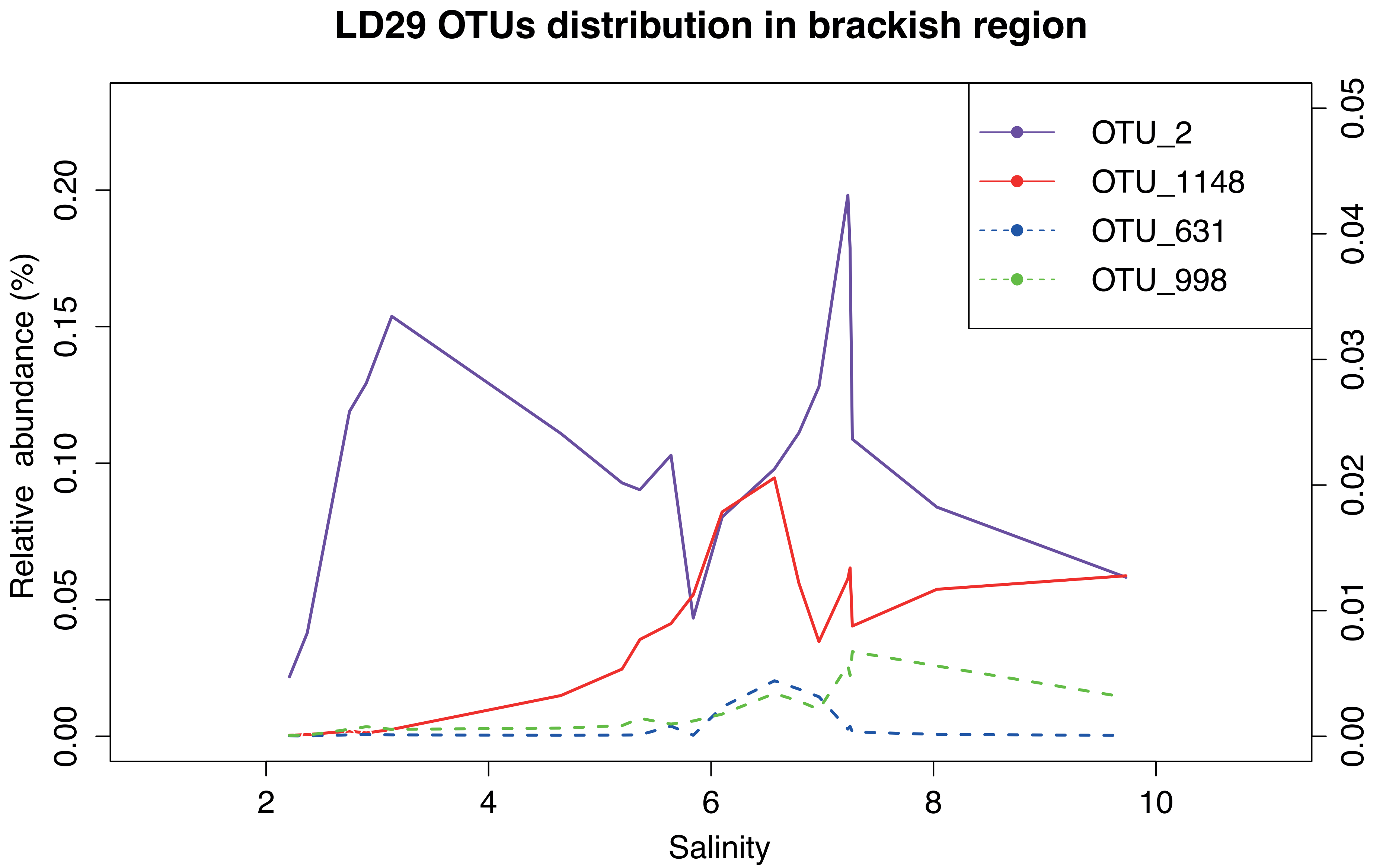

Supplementary figure 5. Distribution of the Candidatus *Spartobacteria baltica* (LD29) OTUs. Left y-axis indicate abundances for OTU_2 and OTU_1148; right y-axis for OTU_631 and OTU_998. (LD29 OTUs displaying > 0.01% mean abundance are shown.)
