# Supplementary material for: Diversity of Pico- to Mesoplankton along the 2000 km Salinity Gradient of the Baltic Sea
Source: Front Microbiol. 2016 May 12;7:679. doi: 10.3389/fmicb.2016.00679 (PMC4864665; doi:10.3389/fmicb.2016.00679)
Supplement: Supplementary file 6 [file Image6.pdf]

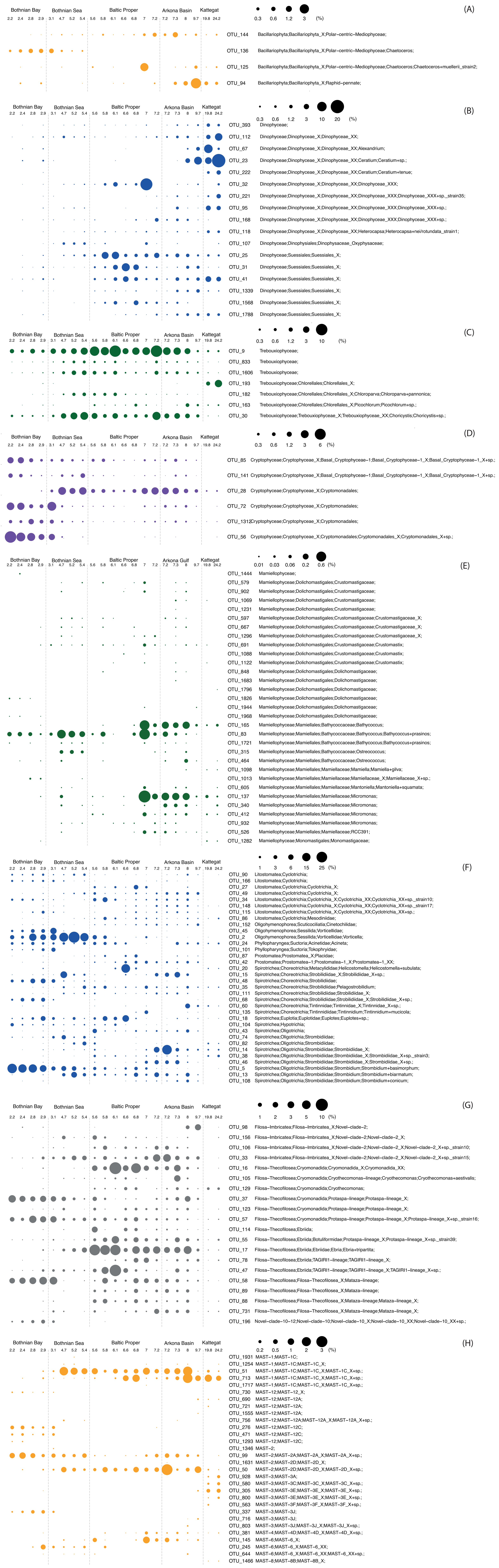

Supplementary Figure 6. Major OTUs in seven protist classes. (A) Bacillariophyta. **(B)** Dinophyceae. **(C)** Trebouxiophyceae. **(D)** Cryptophyceae. **(E)** Mamiellophyceae. **(F)** Ciliophora. **(G)** Cercozoa. **(H)** MAST. OTUs displaying $> 10^{-3}$ mean abundance are shown, except for in **(E)** and **(H)** where all OTUs are shown.
