# Supplementary material for: Diversity of Pico- to Mesoplankton along the 2000 km Salinity Gradient of the Baltic Sea
Source: Front Microbiol. 2016 May 12;7:679. doi: 10.3389/fmicb.2016.00679 (PMC4864665; doi:10.3389/fmicb.2016.00679)
Supplement: Supplementary file 7 [file Image7.pdf]

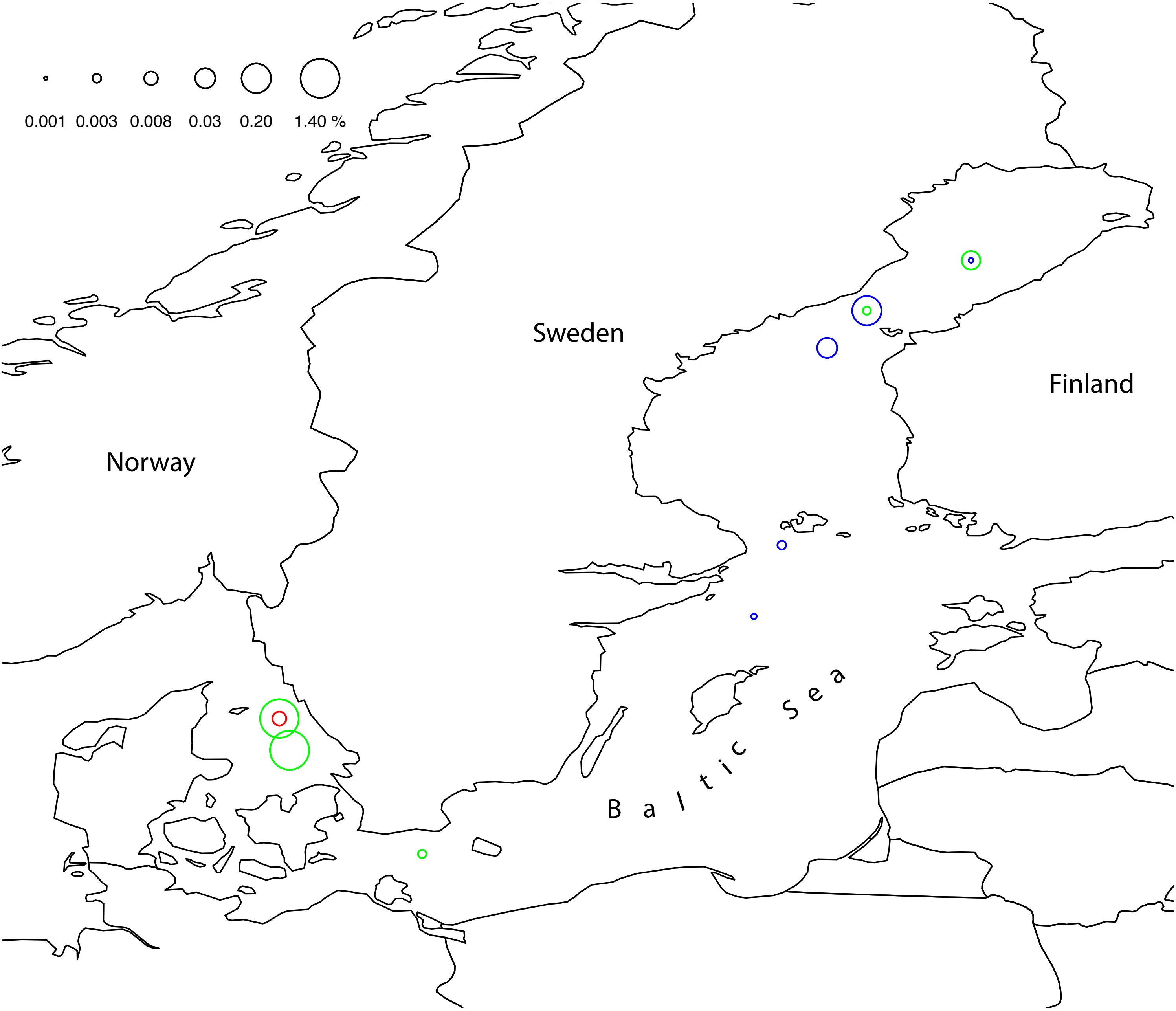

Supplementary figure 7. Relative abundance distribution (in log scale) of the order *Isochrysidales* in the Baltic Sea. Green and red circles show the relative abundance of coccolithophores in log scale (OTU_127, OTU_2007), Blue circles show the non-coccolithophores from the same order (OTU_585). Relative abundances are based on percentages of non-Metazoa reads.
