# Supplementary material for: Diversity of Pico- to Mesoplankton along the 2000 km Salinity Gradient of the Baltic Sea
Source: Front Microbiol. 2016 May 12;7:679. doi: 10.3389/fmicb.2016.00679 (PMC4864665; doi:10.3389/fmicb.2016.00679)
Supplement: Supplementary file 8 [file Image8.PDF]

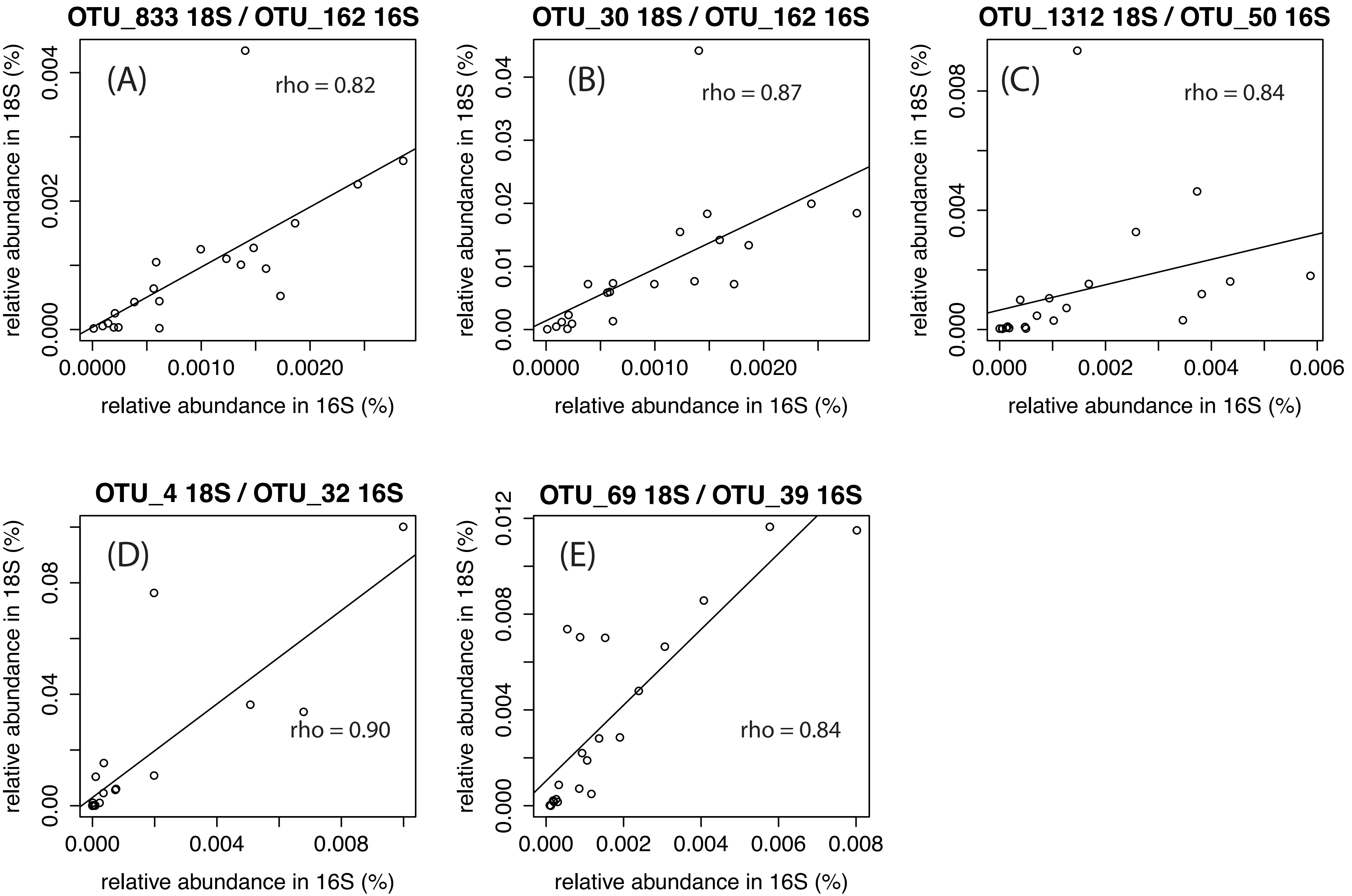

Supplementary figure 8. Correlations of OTU pairs of 16S and 18S OTUs which had matched classification in 16S chloroplast gene and 18S rRNA gene. OTU pairs are from classes **(A,B)** Trebouxiophyceae, **(C)** Cryptophyceae, **(D)** Chrysophyceae, **(E)** Eustigmatophyceae. The Spearman correlation coefficient (rho) is indicated in each plot.
