# Supplementary material for: Diversity of Pico- to Mesoplankton along the 2000 km Salinity Gradient of the Baltic Sea
Source: Front Microbiol. 2016 May 12;7:679. doi: 10.3389/fmicb.2016.00679 (PMC4864665; doi:10.3389/fmicb.2016.00679)
Supplement: Supplementary file 9 [file Table1.PDF]

Salinity	Sampling Date	Sampling Time	Latitude	Longitude	Temperature (°C)	Phycocyanin	Region	Sample ID
2.21	15.07.2013	7:44	65.229	23.774	13.8	0.061	Bothnian Bay (Gulf of Bothnia)	16S_10/18S_10
2.37	16.07.2013	10:49	65.123	24.161	13.3	0.056	Bothnian Bay (Gulf of Bothnia)	16S_11/18S_11
2.75	15.07.2013	4:41	64.643	22.784	13.3	0.048	Bothnian Bay (Gulf of Bothnia)	16S_9/18S_9
2.9	16.07.2013	14:22	64.345	22.507	12.9	0.053	Bothnian Bay (Gulf of Bothnia)	16S_12/18S_12
3.13	16.07.2013	17:47	63.556	20.873	13.7	0.048	Bothnian Sea (Gulf of Bothnia)	16S_13/18S_13
4.65	14.07.2013	20:01	62.973	20.251	15.1	0.048	Bothnian Sea (Gulf of Bothnia)	16S_8/18S_8
5.2	14.07.2013	15:18	61.873	19.718	15.3	0.062	Bothnian Sea (Gulf of Bothnia)	16S_7/18S_7
5.36	14.07.2013	10:18	60.744	19.176	14.5	0.055	Bothnian Sea (Gulf of Bothnia)	16S_6/18S_6
5.64	17.07.2013	6:18	59.88	19.54	14.7	0.078	Baltic Proper	16S_14/18S_14
5.84	14.07.2013	3:15	59.282	19.898	16.9	0.071	Baltic Proper	16S_5/18S_5
6.1	17.07.2013	10:27	58.763	19.103	17.3	0.091	Baltic Proper	16S_15/18S_15
6.57	17.07.2013	15:24	57.427	17.676	17.4	0.053	Baltic Proper	16S_17/18S_17
6.79	13.07.2013	18:00	57.04	17.529	16.8	0.101	Baltic Proper	16S_4/18S_4
6.97	13.07.2013	13:55	56.032	16.65	16.9	0.112	Baltic Proper	16S_3/18S_3
7.23	13.07.2013	9:38	55.434	14.813	17.4	0.147	Baltic Proper	16S_2/18S_2
7.25	13.07.2013	7:08	55.185	13.791	16.9	0.063	Arkona	16S_1/18S_1
7.27	18.07.2013	3:40	55.038	13.901	16.7	0.104	Arkona	16S_18/18S_18
8.03	18.07.2013	6:55	54.765	12.659	18.2	0.11	Arkona	16S_19/18S_19
9.73	18.07.2013	10:37	54.259	11.514	19.1	0.14	Arkona	16S_20/18S_20
19.81	19.07.2013	10:42	56.664	11.822	17.8	0.047	Kattegat	16S_21/18S_21
24.2	19.07.2013	12:23	57.161	11.662	18.3	0.046	Kattegat	16S_22/18S_22

* Phycocyanin Fluorescence was given in arbitrary units.
* Sample IDs are compatible with Sample IDs in OTU tables.
